# Dickkopf Homolog 3 (*DKK3*) Plays a Crucial Role Upstream of WNT/β-CATENIN Signaling for Sertoli Cell Mediated Regulation of Spermatogenesis

**DOI:** 10.1371/journal.pone.0063603

**Published:** 2013-05-07

**Authors:** Deepika Sharma Das, Neerja Wadhwa, Neetu Kunj, Kanchan Sarda, Bhola Shankar Pradhan, Subeer S. Majumdar

**Affiliations:** 1 Division of Cellular Endocrinology, National Institute of Immunology, Aruna Asaf Ali Marg, New Delhi, India; 2 Division of Embryo Biotechnology, National Institute of Immunology, Aruna Asaf Ali Marg, New Delhi, India; University of Hyderabad, India

## Abstract

Testicular Sertoli cells (Sc) are main somatic component of seminiferous tubules that govern the differentiation of germ cells (Gc) and provide them physical support. Sc are the target of follicle stimulating hormone (FSH) and testosterone (T) which are known to regulate spermatogenesis. FSH and T levels in human and sub-human male primates remain high during infancy (4–6 months post birth), similar to those during puberty. Subsequently, juvenile phase is marked with low levels of these hormones. In spite of prolonged hormonal exposure, spermatogenesis is not discerned during infancy unlike that during puberty. Situation during infancy is similar to certain idiopathic male infertility, where prolonged hormone supplementation fails to initiate spermatogenesis. In our quest to determine non hormonal causes of idiopathic infertility which may reside within the Sc, we investigated the association between spermatogenesis and Sc specific gene(s) expressed differentially during puberty and infancy. Although products of several genes may be necessary for quantitatively normal spermatogenesis, one needs to investigate their roles one by one. Differential display and real time PCR analysis revealed higher expression of a known tumor suppressor, Dickkopf homolog 3 (*DKK3*), by pubertal monkey Sc as compared to infant Sc. To evaluate role of *DKK3* in spermatogenesis, we generated *DKK3* knock down mice (DKDM) using shRNA construct targeted to *DKK3*. In testis of adult DKDM, expression of DKK3 mRNA and protein were significantly (p<0.05) low and was associated with elevated WNT-4/β-CATENIN activity. Elevated β-CATENIN activity is known to restrict Sc maturation. Abundant expression of infant Sc marker, Mullerian inhibiting substance (*MIS*), in the testes of adult DKDM confirmed lack of Sc maturation in DKDM. Gc differentiation and fertility was severely compromised in DKDM. This is the first report of role of DKK3 in the testis and DKK3 mediated regulation of spermatogenesis via WNT-4/β-CATENIN modulation.

## Introduction

Sertoli cells (Sc) are the main somatic cells of testis which play a major role in cyto-architectural organization of the seminiferous tubule and most importantly, govern the differentiation of germ cells (Gc). The physical and functional support of Sc is essential for Gc survival and development. Follicle stimulating hormone (FSH) and testosterone (T) are known to regulate spermatogenesis through their receptors present in Sc.

Worldwide, up to 20% of couples are infertile. Approximately 30–50% of human infertility is attributable to male infertility [1], [2]. Although hormonal causes of male infertility are well known, lack of sufficient knowledge about intracellular mechanisms leading to the production of important Sc factors necessary for regulating spermatogenesis is the main reason behind inability to diagnose and treat certain forms of idiopathic infertility. Infancy, in human and non-human primates (up to 4–6 months of age), represents an infertility-like situation because in spite of adequate hormonal levels like those in adults, spermatogenesis is restricted during infancy [3]. Testicular Sc bear receptors for FSH and T through which these cells modulate expression of their genes and gene products which are necessary for spermatogenesis within seminiferous tubule, during pubertal and adult phase of life [4]. We have recently shown that functional ability of infant Sc is inadequate as compared to pubertal Sc in rats [5] and sub-human primates [6]. Hence, comparative evaluation of genes expressed by infant and pubertal Sc exposed to identical hormonal milieu may lead to identification of gene(s) or gene product(s) relevant to onset of robust spermatogenesis during normal puberty but not during infancy. Although involvement of multiple genes may not be ruled out, stepwise progress in divulging their role(s) one by one may be necessary for laying strong foundation to address causes of idiopathic infertility.

Expression of DKK3, which belongs to the Dickkopf (*DKK*) family of genes, which are known to regulate Wnt signaling, was found to be augmented remarkably in pubertal monkey Sc as compared to infant Sc. A recent study suggested that diminished WNT/β-CATENIN activity in Sc during pubertal development allows functional maturation of Sc which enables them to express genes conducive to spermatogenesis [7]. We hypothesized that *DKK3* may be one of the factor(s) which presumably regulate WNT/β-CATENIN activity in testis, hence, may have a crucial role in causing maturity of Sc leading to onset of spermatogenesis.

Testis is an organ where cell division and differentiation, a phenomenon known to be regulated by WNT signaling, continues throughout life. However, association of the components of WNT signaling with testicular Gc differentiation has not been extensively studied, although WNT signaling has been shown to play an important role in proliferation and self-renewal of mouse and human spermatogonia [7], [8].

Constitutively activated form of β-CATENIN in mouse Sc is known to keep them in an immature state even during adulthood [7] and is known to interrupt male fertility via progressive degeneration of seminiferous tubules and testicular atrophy associated with loss of Gc [9]. These observation suggested that altered WNT/β-CATENIN signaling inhibits postnatal differentiation of Sc, hampering attainment of their functional ability to regulate Gc division and differentiation causing increased Gc apoptosis and infertility [7], [10]. On this basis, it is reasonable to assume that during normal course of development, reduction in WNT/β-CATENIN activity during pubertal development allows Sc to mature and support spermatogenesis at puberty. However, the factor which leads to such reduction in WNT/β-CATENIN activity and which is crucial for Sc maturation during puberty is yet unknown.

Although role of *DKK3* in regulating WNT signaling was not clear [12]–[14], recently *DKK3* is shown to inhibit canonical WNT signaling in lung and breast cancer cells [15]. Since, involvement of *DKK3* in targeting WNT pathway in cell specific manner is gaining momentum [16], present study was designed to explore whether elevated expression of *DKK3* in Sc during puberty is responsible for inhibiting WNT/β-CATENIN signaling, which is known to induce Sc maturity and thereby enhance their ability to generate sperm. We found that *DKK3* regulates Sc maturation through inhibition of WNT-4/β-CATENIN signaling.

## Materials and Methods

### Ethics statement

All experimental animals (monkeys as well as mice) were kept and used as per the National guidelines provided by the Committee for the Purpose of Control and Supervision of the Experiments on Animals (CPCSEA) in India. All animal experiments in this study were performed following protocols approved by the Institutional Animal Ethics Committee (IAEC Number 49/99, 187/08, 249/10) of the National Institute of Immunology (New Delhi, India). FVB/J mice were procured from the Small Animal Facility of the National Institute of Immunology. When required, some of the FVB/J mice were sacrificed by cervical dislocation, as approved by the Institutional Animal Ethics Committee of National Institute of Immunology. Rhesus male monkeys (*Macaca mulatta*) born and raised at the Primate Research Center of National institute of immunology were used for this study. The selected monkeys were members of captive breeding group that lived within open enclosures enriched with swings and perch and consumed fruits, soaked gram and pelleted feed (Golden feed, Delhi, India). Prior to catheterization or castration of monkeys, they were anesthetized with pentobarbital sodium (∼25 mg/kg body weight, i.v., plus 5 mg supplements as required). Postoperatively, each monkey received cefotaxime (75 mg/kg body weight) and an analgesic, diclofenac sodium (1 mg/kg body weight), i.m., twice daily for 5 days. No monkey was sacrificed for this study.

### Preparation of pubertal monkeys

Precocious puberty associated with robust Gc differentiation was induced in juvenile monkeys (18–22 months) by activation of the dormant pituitary-testicular axis using pulsatile GnRH (0.3 µg GnRH/2 ml saline/2 minutes/3 hours) treatment for 4–5 weeks as reported by us previously [17]. Briefly, juvenile male monkeys were surgically implanted with chronic indwelling catheters via femoral or internal jugular vein under sterile conditions. The catheter was exteriorized in the midscapular region. The exteriorized catheter was protected by a nylon jacket and a flexible stainless steel tether (36 inches long, 0.5 inches inner diameter) attached to a swivel device on top of the cage. This system allowed normal free movement of the monkeys without affecting continuous access to venous circulation via the catheter. Monkeys were treated with intermittent pulsatile GnRH for 4–5 weeks, until serum T levels reached and were maintained for a week in the adult range. Weekly blood samples were collected via catheter to measure the circulating levels of T, before and after the GnRH pulse.

### Isolation and culture of Sc

Testes from infant (3 months old) and pubertal monkeys were surgically removed under general anesthesia [17] and Sc were isolated and cultured following the procedure previously described by us [17], [18]. Similarly, Sc were cultured from 7 days and 20 days old FVB/J mice according to the procedure described by Welsh and Wiebe [19] with minor modifications. On day 4 of culture, hypotonic shock was given for 3 minutes to remove Gc, if any in the culture and on day 5 of culture, Sc were treated with rmFSH (5 ng/ml) and T (10^−7^ M) in combination for 24 hours to mimic *in vivo* situation.

### Differential display analysis of monkey Sc

To study differential gene expression between infant and pubertal Sc, RNA from cultured cells were extracted after 24 hour of hormone treatment using trizol and cDNA were synthesized to perform differential display procedure as per the established method [20]. PCR was performed using 48 short arbitrary primers. PCR products were separated on 6% urea sequencing gels. Differentially amplified bands were selected, cloned into PCR-TRAP vector (GenHunter, Charlottesville, VA, USA) amplified and sequenced. For identification of nucleotide sequences homologous to differentially expressed bands, BLAST program was used.

### Designing of DKK3 shRNA knock down construct and generation of *DKK3* knock down mice (DKDM)

DNA sequences encoding shRNA specific to *DKK3* were synthesized and cloned into pRNAT-CMV3.1/Neo vector (GenScript, NJ, USA) between BamHI and AflII sites. shRNA sequences for knocking down *DKK3* were as follows:

#### Forward oligos

GATCGTACCAATTGGCAGGAAGTTCACAAGATAACCAATCAAGAGTTGGTTATCTTGTGAACTTCCTGTTTTTTCAATTGGTAC;

#### Reverse oligos

TTAAGTACCAATTGAAAAAACAGGAAGTTCACAAGATAACCAACTCTTGATTGGTTATCTTGTGAACTTCCTGCCAATTGGTAC.

Positive clones were confirmed by sequencing. The shRNA vector comprised of *CMV* promoter which drove the expression of shRNA and *SV40* promoter which drove the expression of the *GFP*. Positive clones were confirmed by sequencing. shRNA clones were linearized with SalI and 4 kb fragment was eluted. *DKK3* knock down mice (DKDM) were generated using the procedure of testicular electroporation as described by us previously [21]. Similarly, FVB/J mice were also electroporated with construct in which scrambled sequences of DNA similar but not identical to those encoding *DKK3* shRNA were cloned. Pups born were analyzed for shRNA construct integration by normal PCR. Genotyping was performed using DNA from tail biopsies of 3 weeks old mice, using standard PCR protocols and transgene specific primers. Tail biopsies (3 mm) were lysed for 16 hours at 55°C in high salt digestion buffer containing 50 mM Tris HCl, 1% SDS, 100 mM NaCl, 100 mM EDTA and 1200 µg/ml Proteinase K. The lysate was processed for isolation of DNA using phenol-chloroform extraction followed by ethanol precipitation. Extracted genomic DNA (gDNA) was subjected for PCR analysis using primers sequences as follows: forward 5′-GCCCCATGGCTGACTAATTT-3′; reverse 5′- GTATCGCCCTCGAACTTCAC-3′. Forward primer was designed to recognize sequences on SV40 promoter and reverse primer was designed to recognize sequences on GFP. Every PCR reaction set had two controls. PCR of *DKK3* shRNA construct was used as a positive control, PCR of gDNA obtained from WT mice was used as a negative control. The PCR reaction was performed using Perkin Elmer Thermal Cycler. Reaction conditions were as follows: 94°C for 5 minutes followed by 30 cycles of 94°C for 30 seconds, 60°C for 30 seconds and 72°C for 30 seconds. The product of 500 bp was visualized on 1% agarose gel with ethidium bromide. PCR analysis till F4 generation was done to confirm the heritable genomic integration. For Slot-Blot analysis, 1 µg gDNA samples were blotted onto a nitrocellulose membrane using a slot blotter. Pre-hybridization, hybridization with probe and washings were done following standard procedure [22]. Kodak biomax film was exposed to hybridized membrane at -70°C for 48 hours. The probe used was the 4 kb SalI fragment of pRNAT-CMV3.1 Neo vector containing *DKK3* shRNA sequence.

### Real Time PCR analysis

Infant and pubertal monkey Sc treated with rmFSH and T for 24 hours *in vitro* were used to extract RNA. RNA was also isolated from cultured Sc of 7 days old (infant) and 20 days old (pubertal) FVB/J mice. Real time PCR was performed to detect expression of *DKK3*, Mullerian inhibiting substance *(MIS), β-CATENIN* and different Wingless related protein (*WNT*) genes at ten weeks of age. Real time PCR of *DKK3* and *β-CATENIN* was also carried out using RNA isolated from purified cultures of Sc. Testes from DKDM and wild type (WT) mice were surgically dissected and RNA was extracted using TRIzol. Real time PCR was performed using different primers specific for respective genes. The list of genes along with primer sequences are given in Table S1. RNA (1 µg) was treated with DNase I (1 µg) for 15 minutes at 25°C. Reaction was terminated by adding 1 µl of 25 mM EDTA and incubating at 65°C for 10 minutes. DNaseI treated RNA was reverse transcribed using Reverse Transcription System (Promega Corp, USA) with AMV reverse transcriptase and oligo (dT)_15_ for the single-strand cDNA synthesis. Real time PCR amplifications were performed in optical tubes in the Realplex (Eppendorf, Hamburg, Germany) in a total volume of 10 µl, which included 1 µl of cDNA and 5 µl of Power SYBR Green Master Mix (Applied Biosystems, CA, USA). CyclophillinA was used as an endogenous housekeeping gene control. Differences in relative expression levels was derived from 2^−ΔCt^ method, where ΔCt  =  Ct gene of interest - Ct internal control as described by Schmittgen and Livak [23].

### Histological analysis, immunohistochemistry and TUNEL staining

Tissue histology was performed as described by us previously [17]. Briefly, mice testes were immersion fixed in Bouin's fluid for 10–12 hours at room temperature and processed for making tissue blocks before sectioning them at 4 µm thickness. Sections were stained with hematoxylin and eosin for evaluating the status of spermatogenesis. Seminiferous tubular diameter was measured at 20× magnification using measuring eyepiece. Only round and oval tubules were considered for diameter measurement. The numbers of normal and degenerated tubules were also counted under equal area of observation (per field) at magnification 20×. Tubules showing loss of Gc, sloughing off of Gc and presence of vacuoles were considered as degenerated tubules. Immunohistochemical studies were performed as described previously [7]. Following primary and secondary antibodies were used: rabbit anti-mouse DKK3 (Santa Cruz Biotechnology, CA,USA), goat anti-mouse MIS (Santa Cruz Biotechnology, CA,USA), mouse anti-GFP (Abcam, Cambridge, MA,USA), β-CATENIN anti-mouse antibody (Santa Cruz Biotechnology, CA,USA), Alexa Fluor 488 anti-mouse secondary antibody (Invitrogen, Life Technologies, NY, USA), FITC anti-rabbit secondary antibody (Abcam, Cambridge, MA,USA), Cy5 anti-goat antibody F(ab)2 (The Jackson ImmunoResearch Laboratory, PA,USA). TUNEL staining was performed according to the manufacturer's recommended protocol (Roche Applied Science, Indianapolis, IN,USA). Testicular paraffin sections were deparaffinized, rehydrated by successive serial washings with ethanol and treated with proteinase K for permeabilizion of cells. Fragmented DNA was labeled with terminal deoxynucleotidyl transferase and biotin dNTPs. The streptavidin-horseradish peroxidase and diaminobenzidine tetra hydrochloride system was used to visualize for the apoptotic cells under light microscope. Quantification of apoptotic germ cells in WT and DKDM was done and compared by observing random visual fields under equal area of observation (per field) under magnification 20×. Immunohistochemistry images were photographed by using Olympus IX81 microscope equipped with fluo view SV1000. Histology and TUNEL images were photographed with DS-5M camera assisted with Digital sight DS-LI software. For immunocytochemistry analysis, Sc were cultured on coverslips for 4 days and then washed in PBS before being fixed in 2% paraformaldehyde for 15 minutes. Cells were permeabilized in 0.1% Tween 20 prior to blocking with goat serum and then incubated with mouse antiserum raised against β-CATENIN for 2 hours at room temperature. Bound primary antisera were detected by secondary antibodies tagged with Alexa 488. Sc nuclei were stained with DAPI. Immunocytochemistry images were captured on confocal microscope (Nikon, A1Rsi).

### Assessment of serum testosterone levels and testosterone replacement

Blood serum testosterone (T) level of juvenile monkey was measured before and after the GnRH pulse as described by us [17]. Serum T levels from the DKDM and WT mice were assessed at ten weeks of age. Blood was obtained through retro-orbital bleeding before sacrificing the mice. Serum T levels was assayed by RIA in duplicate. The intra- and inter assay coefficients of variation were less than 7% and less than 10%, respectively [17]. For T replacement, 20 µl (250 mg/ml) of testosterone undecanoate (TU) was injected once intramuscularly to each DKDM as reported for mice [24]. Additionally, WT and DKDM mice were injected with 20 µl of castor oil which was used as vehicle. Blood was taken retro-orbitally after a month of injection and serum T levels were measured.

### Immunoblot analysis

Testicular fraction from WT and DKDM were lysed separately with ice-cold PBS (pH 7.4) containing 50 mM Tris Chloride, 150 mM NaCl, 1% Triton ×100 and protease inhibitors (1 mM PMSF, 1 µg/ml aprotinin and 1 µg/ml leupeptin). Lysates were centrifuged (13,000× *g*, 4°C, 30 minutes) and supernatant were stored separately at 80°C, until immunoblot analyses for DKK3 and MIS were performed as described previously [25]. Protein concentrations were determined by the Bradford method. 20 µg protein from supernatant was resolved by one-dimensional SDS-PAGE (12% acrylamide) under reducing conditions and electrophoretically transferred to a nitrocellulose membrane. After blocking with blotto (Santa Cruz, CA, USA), the membranes were incubated with primary antibody (1∶500) in blocking buffer at 4°C for overnight and then with the goat anti-rabbit or rabbit anti-goat horseradish peroxidase-conjugated secondary antibody (1∶5000) for 2 hours at room temperature. After washing the blot thrice in PBS (1×), protein bands were visualized by chemiluminescence using the ECL plus western Blotting Detection Reagents (GE Amersham, Piscataway, NJ, USA) and chemiluminescence film (GE Amersham, Piscataway, NJ, USA).

### Testicular weights, sperm counts and fertility assessment

Testes of both WT and DKDM were removed, weighed and recorded at the age of ten weeks. Testis weight per gram body weight was also evaluated. The concentration of epididymal spermatozoa and the litter size from the productive matings was analyzed. Total number of sperm present in both epididymis of each mouse was counted after releasing the sperm in 1 ml of PBS by puncturing epididymus at several sites and squeezing. The total number of sperm was determined by using a hemocytometer. DKDM male and female from F1 generation (siblings) were cohabitated for three weeks. This ensured exposure of female to the male at least through five ovarian cycles. Litter size was determined after delivery of progeny to assess fertility of parents. Similarly, WT mice were also assessed for fertility.

### Statistical Analysis

Data was analyzed using Mann–Whitney test. All statistical analyses were performed using Graphpad Prism 4.01,(Graphpad Software, Lajolla, CA, USA). Data is represented as means ± SEM of atleast three animals belonging to a particular group. Significance was determined at p<0.05.

## Results

### Differential gene expression by infant and pubertal Sc

**Figure 1:**
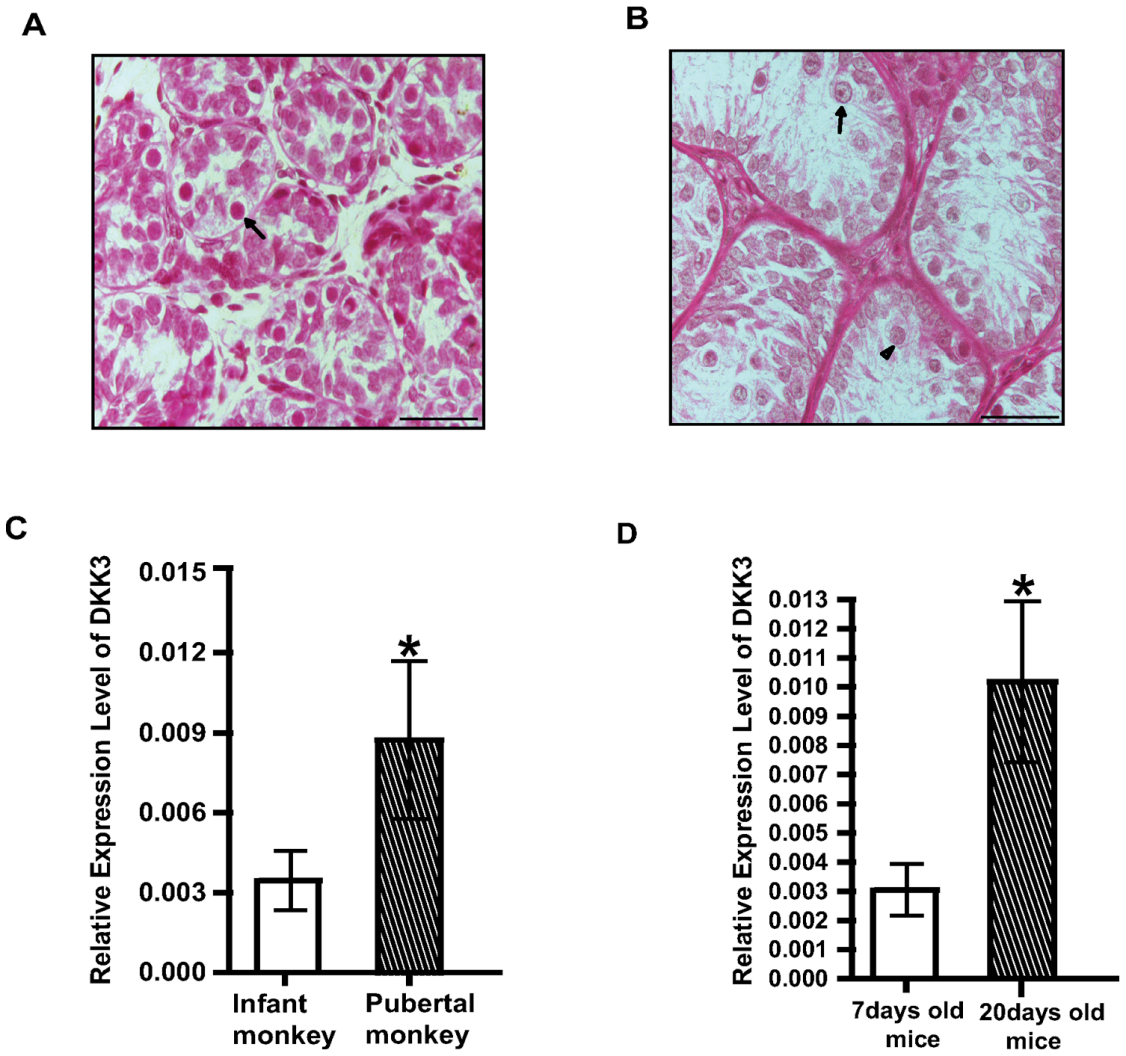
*DKK3* is over expressed in Sc during puberty of monkeys and mice. (A) Testicular sections showing seminiferous tubules of an infant monkey. Spermatogonia A are shown with arrow. Scale bar: 50 µm (B) Testicular sections showing seminiferous tubules of pubertal monkey. Spermatogonia B (shown with arrow) and spermatocytes (shown with arrowhead) can be noticed in pubertal monkey. Scale bar: 50 µm (C) Relative quantity of *DKK3* mRNA expression in Sc from infant (open bar) and pubertal monkeys (hatched bar). Real time PCR data from Sc of three animals are represented as mean +/− SEM in each bar (*p<0.05). (D) Relative quantity of *DKK3* expression in Sc from 7 days old (open bar) and 20 days old mice (hatched bar). Real time PCR data from Sc of three animals are represented as mean +/− SEM in each bar (*p<0.05).

Puberty, known to occur at around 3.5 years of age in rhesus monkeys which is associated with surge in gonadotropins & T resulting into division and differentiation of spermatogonial cells. Since it is difficult to pinpoint the onset of puberty, juvenile male monkeys were treated with pulsatile GnRH to induce puberty in them, as described previously by us [17]. T levels in such monkeys were increased by 6–10 fold, testicular weight were increased by 5–6 fold and markers of initiation of spermatogenesis such as enlarged seminiferous tubules containing large populations of spermatogonia B and primary spermatocytes were evident (Fig. 1A and B) like those during puberty [26].

Sc cultured from infant (3 months old) and pubertal monkeys were 95% pure, as seen by oil Red O positive cells. To ascertain differential gene expression by immature and mature Sc, RNA was isolated from cultured Sc and cDNA was synthesized to perform fluorescent differential display (Fig. S1A). Differentially expressed sequences (infant vs. pubertal) were analyzed and matched with homologous primate-specific gene sequences. Expression of *DKK3* was elevated (more than five folds) in pubertal Sc as compared to infant Sc. This was reconfirmed by real time PCR analysis using mRNA obtained from cultured Sc from infant and pubertal monkeys (Fig. 1C). Additionally, Sc cultured from 20 day old (spermatogenically active) mice also showed significantly (p<0.05) higher expression of *DKK3* mRNA as compared to Sc from 7 day old (spermatogenically quiescent) mice (Fig. 1D). This provided strong basis to generate transgenic knock down mice for evaluating spermatogenic role of *DKK3 in vivo*.

### Generation and analysis of *DKK3* shRNA knock down mice

**Figure 2:**
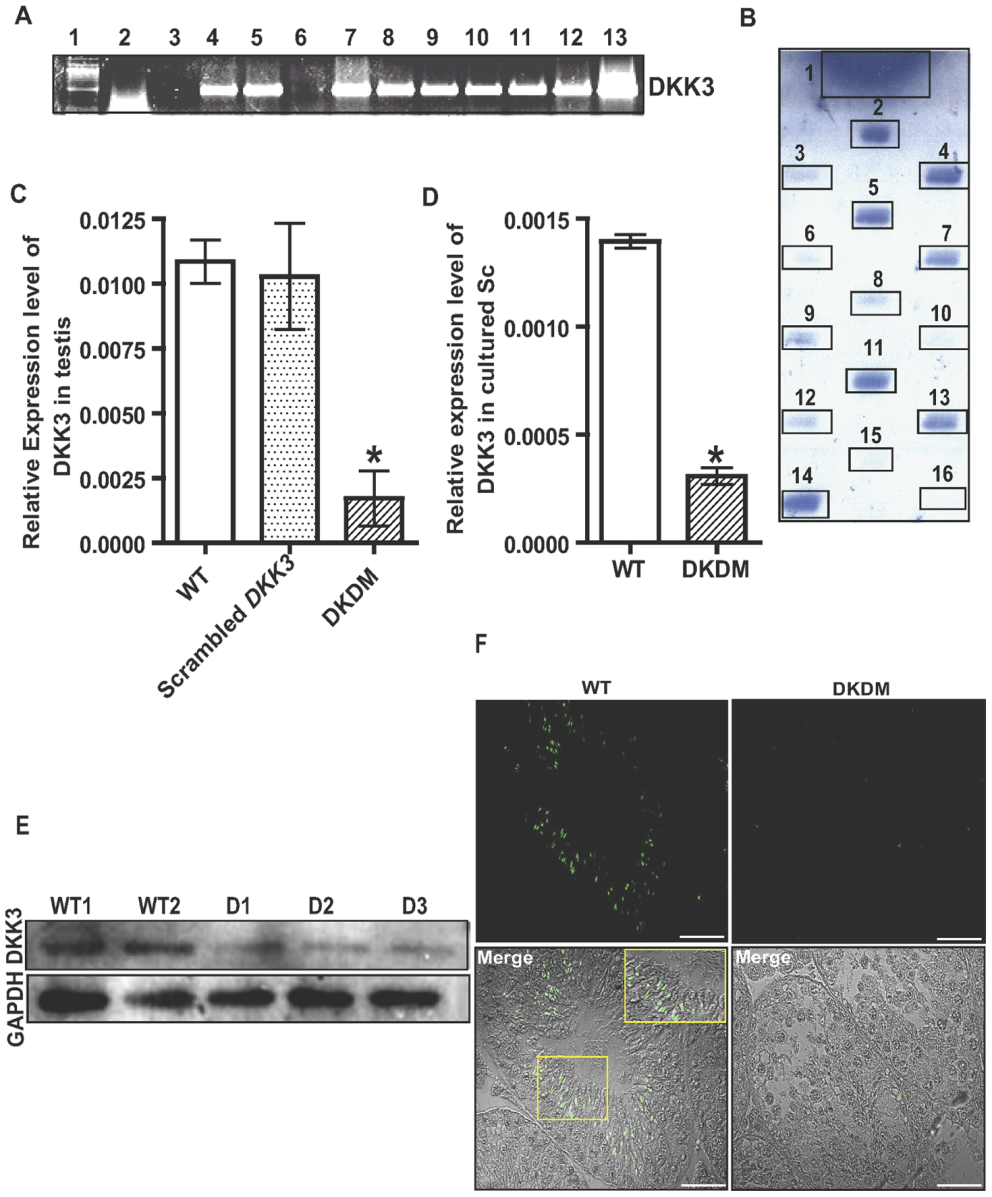
Characterization of DKDM. (A) PCR results using gDNA obtained from tail biopsies of progeny generated by crossing electroporated male and WT female mice. Lane 1: 100 bp marker, Lane 2: gDNA of WT mice, Lane 3–12: gDNA of F1 progeny, Lane 13: *DKK3* shRNA construct. (B) Slot Blot analysis of PCR positive samples of F1 progeny. DNA samples of PCR positive and WT mice (negative control) were hybridized with transgene specific probe. *DKK3* shRNA construct was used as a positive control. Sample 1 - *DKK3* shRNA construct, samples 2–14 - gDNA from PCR positive mice, samples 15 and 16 - gDNA from two different WT mice. (C) Real time PCR of *DKK3* mRNA in the testes of WT mice (open bar), scrambled *DKK3* (dotted bar) and DKDM (hatched bar) at ten weeks of age. Data are represented as mean +/− SEM (*p<0.05, n = 3). (D) Relative quantity of *DKK3* mRNA expression in Sc from WT mice (open bar) and DKDM (hatched bar). Real time PCR data of Sc isolated from four or more individual mice of each group is represented as mean +/− SEM (*p<0.05). (E) Western blot analysis of DKK3 from the testes of WT mice and DKDM at ten weeks of age. WT1 and WT2 represents testicular lysates from two different wild type mice, D1–D3 represents testicular lysates from three individual DKDM. The quantity of Glyceraldehyde-3-phosphate dehydrogenase (GAPDH) used as a housekeeping gene is shown in the lower panel. Note: equal amount of protein was loaded in each well. (F) Immunohistochemical localization of DKK3 in testicular sections showing fluorescence and merged images of WT mice (showing higher expression of DKK3) and F1 generation of DKDM (showing diminished expression of DKK3) at ten weeks of age, Scale bar: 50 µm. Inset in the merged image of WT mice shows magnified area of the boxed region. All these images are representatives of atleast three random visual fields obtained from atleast three or more animals of each group (WT and DKDM).

The DNA encoding shRNA sequences targeting *DKK3* gene were designed and cloned in pRNAT-CMV 3.1/Neo vector (Fig. S1B). This construct was used for *in vivo* electroporation in the testis of FVB/J mice, to generate transgenic mice by electroporation. Similarly, FVB/J mice were also electroporated with construct having scrambled sequences. Pups born from the mating of such electroporated males and WT females were screened for the transgene by PCR of gDNA obtained from tail tissue of the progeny. Majority of the pups born were PCR positive (Fig. 2A). Slot-blot hybridization with *DKK3* shRNA specific probe confirmed the genomic integration of shRNA vector in most of the PCR positive mice (Fig. 2B). RNA extracted from testes of such mice was analyzed quantitatively by real time PCR for determining expression of *DKK3* gene in comparison to that of age matched WT mice. Expression of *DKK3* significantly (p<0.05) declined in *DKK3* knock down mice (Fig.2C), such mice were referred as DKDM. A range of 40%–96% reduction was seen in the expression of *DKK3* in F1 progeny as compared to WT controls (Fig. S2G). Mice generated from different sperm of a founder (electroporated male) displayed a range of different *DKK3* expression based on the differential extent of inhibition of *DKK3* mRNA and were associated with oligo or azoospermia (Fig. S2G). Suppression of *DKK3* was not found in any of the mice generated using scrambled shRNA construct (Fig. 2C). Significant (p<0.05) reduction in the levels of DKK3 were also found in Sc isolated from DKDM in comparison to Sc isolated from WT mice (Fig. 2D). Western blot analysis performed using protein extracts from DKDM testes and their age-matched WT control mice revealed a remarkable decline in DKK3 in DKDM (Fig. 2E). Testes of DKDM expressed GFP which was used as a reporter in the construct (Fig. S1C). The shRNA mediated knock down effect in seminiferous tubules of DKDM was confirmed by immunohistochemistry which showed reduced levels of DKK3 protein in comparison to to WT (Fig. 2F).

### Compromised fertility in DKDM

**Figure 3:**
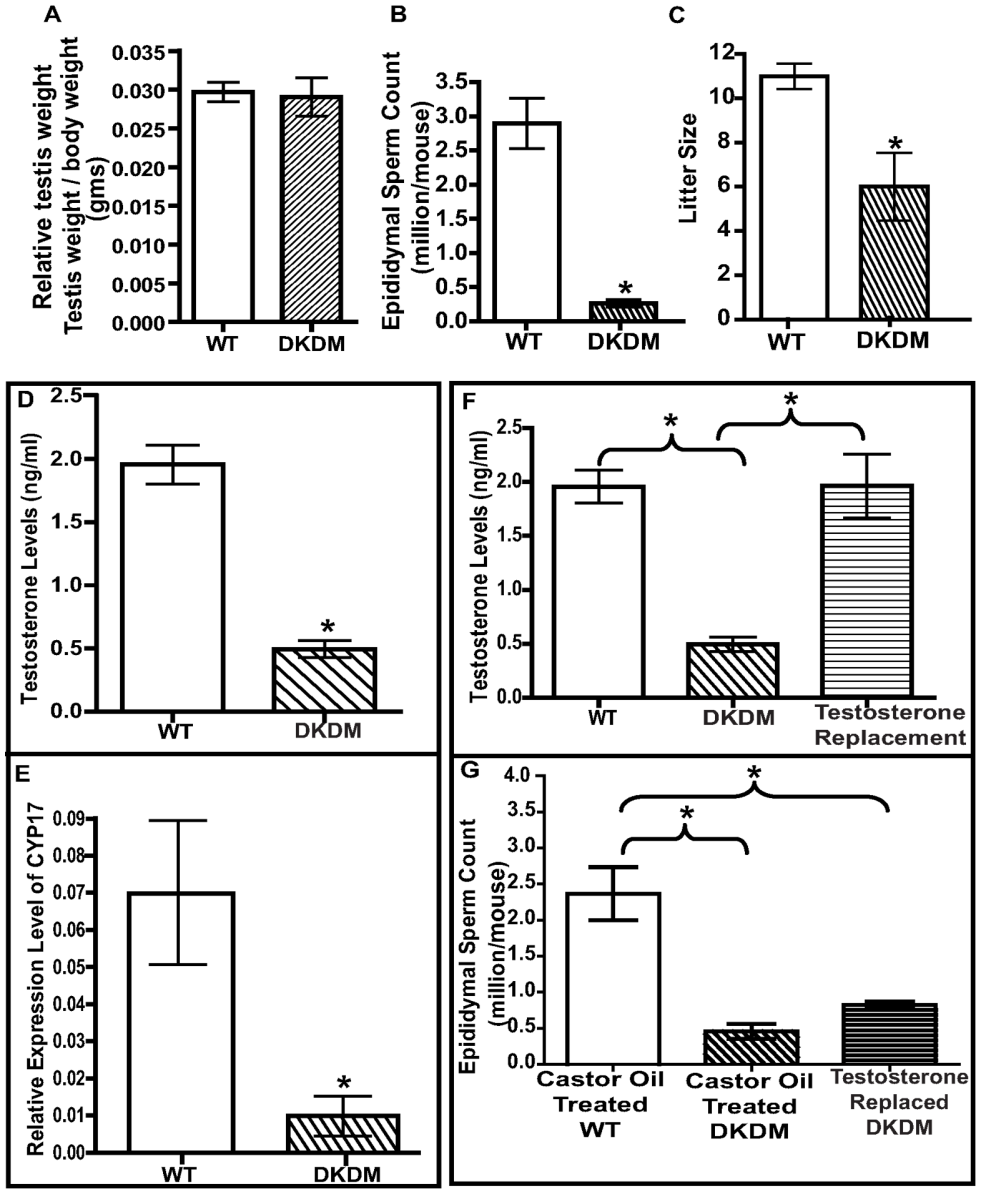
Reduction in *DKK3* leads to impaired spermatogenesis and reduced fertility. (A) Relative testis weight (testis weight per gm body weight) of WT mice (open bar) and DKDM (hatched bar) at ten weeks of age. Data are represented as mean +/− SEM (*p<0.05, n = 10). (B) Mean epididymal sperm count of WT mice (open bar) and DKDM (hatched bar) at ten weeks of age. Data are represented as mean +/- SEM. (*p<0.05, n = 10). (C) Mean litter size of WT mice (open bar) and DKDM (hatched bar). Data are represented as mean +/− SEM (*p<0.05, n = 4). (D) Mean serum T levels of WT mice (open bar) and DKDM (hatched bar). Data are represented as mean +/− SEM. (*p<0.05, n = 3). (E) Relative quantity of *CYP17* mRNA levels in the testes of WT mice (open bar) and DKDM (hatched bar) at ten weeks of age. Real time PCR data from the testicular samples of three animals are represented as mean +/− SEM in each bar (*p<0.05). (F) Serum T levels of WT mice (open bar), DKDM (hatched bar) and T replaced DKDM (horizontal hatched bar). Data are represented as mean +/− SEM (*p<0.05, n = 3). (G) Mean epididymal sperm count of vehicle (castor oil) treated WT mice (open bar), vehicle (castor oil) treated DKDM (hatched bar) and T replaced DKDM (horizontal hatched bar). Data are represented as mean +/− SEM (*p<0.05, n = 3).

Gross morphological assessment of the testes of F1 generation of DKDM, at ten weeks of age showed atrophy of the testes with a significant (p<0.05) reduction in testis weight (Fig. S2F) as well as relative testicular weight per gram body weight (Fig. 3A, Fig. S2E). DKDM displayed drastic (p<0.01) reduction in sperm counts as compared to their age-matched WT mice (Fig. 3B). Mating between DKDM siblings (of F1 generation) resulted in more than 50% reduction in litter size as compared to WT controls (Fig. 3C). Interestingly, mouse showing more than 90% of *DKK3* inhibition, whether in F1 generation or in F4 generation had azoospermia (Fig.S2G). Reproductive functions of mice generated using scrambled sequences for shRNA were not different from WT mice; their F1 progeny were as fertile as WT and they did not show any reproductive defects (Fig. S2A-D). “Hence for all further studies, data from WT mice were used for comparisons”.

Litter size of *DKK3* female when mated with WT male was normal and similar to that generated by the mating between WT male and female (Fig S3B). In contrast, male DKDM generated significantly (p<0.05) low litter size, upon mating with WT females (Fig.S3B).

**Figure 4:**
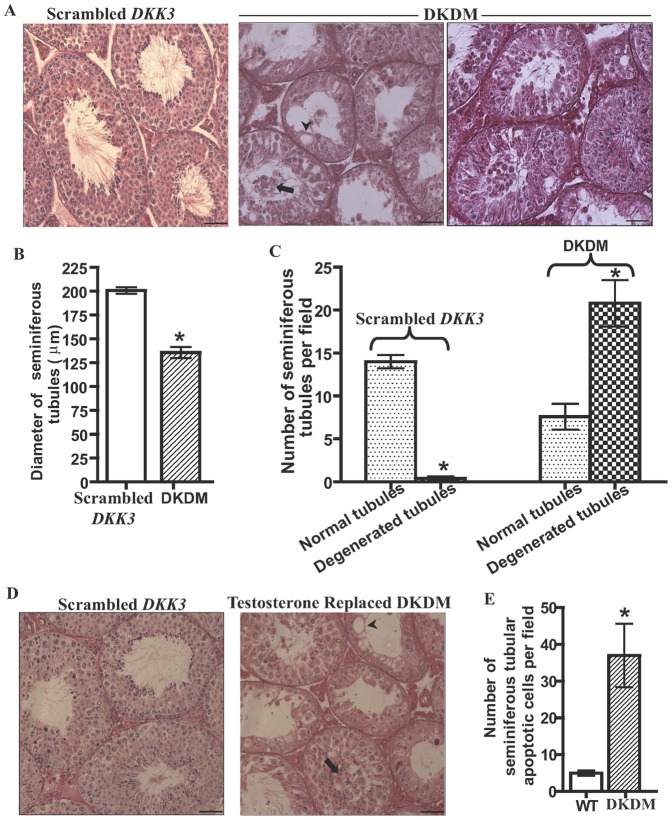
Reduction in *DKK3* causes disruption of seminiferous tubules. (A) Seminiferous tubules of ten weeks old DKDM showing sloughing of Gc (shown by arrow), giant vacuoles (shown by arrowhead) and tubular degeneration. In some of the tubules, sperm were also present. However, the age matched scrambled *DKK3* mice showed normal spermatogenesis. Scale bar: 50 µm. (B) Seminiferous tubular diameter (in µm) of control mice generated using scrambled *DKK3 shRNA* construct (open bar) and DKDM (hatched bar) at ten weeks of age. Round and oval tubules were considered for plotting data from three individual mice of each group under equal area of observation (per field) as seen under magnification 20×. Data is represented as mean +/− SEM in each bar. (*p<0.05). (C) Number of normal tubules (dotted bar) and degenerated tubules (crossed bar) in control mice generated using scrambled *DKK3 shRNA* construct and DKDM at ten weeks of age. Data is plotted by counting normal and degenerated tubules of three individual mice of each group under equal area of observation (per field) as seen under magnification 20×. Data is represented as mean +/− SEM in each bar. (*p<0.05). (D) Seminiferous tubules of ten weeks old control mice generated using scrambled *DKK3 shRNA* construct and DKDM supplemented with T. T replaced DKDM did not show restoration of normal spermatogenesis in seminiferous tubules. Sloughing of Gc (shown by arrow), giant vacuoles (shown by arrowhead) and tubular degeneration was still seen, Scale bar: 50 µm. All these images are representatives of atleast three random visual fields obtained from atleast three or more animals of each group (Scrambled, DKDM and T replaced DKDM). (E) Quantification of apoptotic Gc in the seminiferous tubules of WT mice and DKDM at ten weeks of age. Data is plotted from random visual fields obtained from atleast three or more individual mice of each group under equal area of observation (per field) as seen under magnification 20×. Data is represented as mean +/− SEM in each bar. (*p<0.05).

We found that serum levels of T were significantly (p<0.05) reduced in DKDM as compared to age-matched WT mice, at ten weeks of age (Fig. 3D). This was associated with significantly (p<0.05) low level of Cytochrome p450c17alpha (*CYP17*) mRNA (Fig. 3E). Despite low levels of T, most of the heterozygous DKDM were fertile, although litter size was reduced. To evaluate the relative contribution of low levels of T to the observed decline in the sperm count, we supplemented T levels in DKDM. One month post-treatment with long acting testosterone undecanoate (TU), serum T levels of DKDM reached similar to levels found in WT mice but failed to restore sperm counts. (Fig. 3F and 3G). Histological evaluation of testes from F1 generation of DKDM revealed reduction in tubular diameter showing signs of degeneration with the presence of vacuoles and sloughing of Gc in degenerated tubules (Fig. 4A and 4B). Some of the tubules with normal spermatogenesis were also present in DKDM. The degenerative process was observed in up to 50–80% of tubules (Fig. 4C). Restoration of T levels by TU treatment failed to restore normalcy of seminiferous tubules in DKDM (Fig. 4D).

### Reduced *DKK3* and Gc apoptosis

In order to assess whether defective differentiation of Sc in DKDM caused Gc apoptosis, we performed TUNEL analyses and counted TUNEL-positive cells. Higher numbers of apoptotic cells were observed in degenerated tubules of DKDM testes as compared to that in WT mice (Fig. S2H and 4E).

### Persistent WNT signaling in DKDM

**Figure 5:**
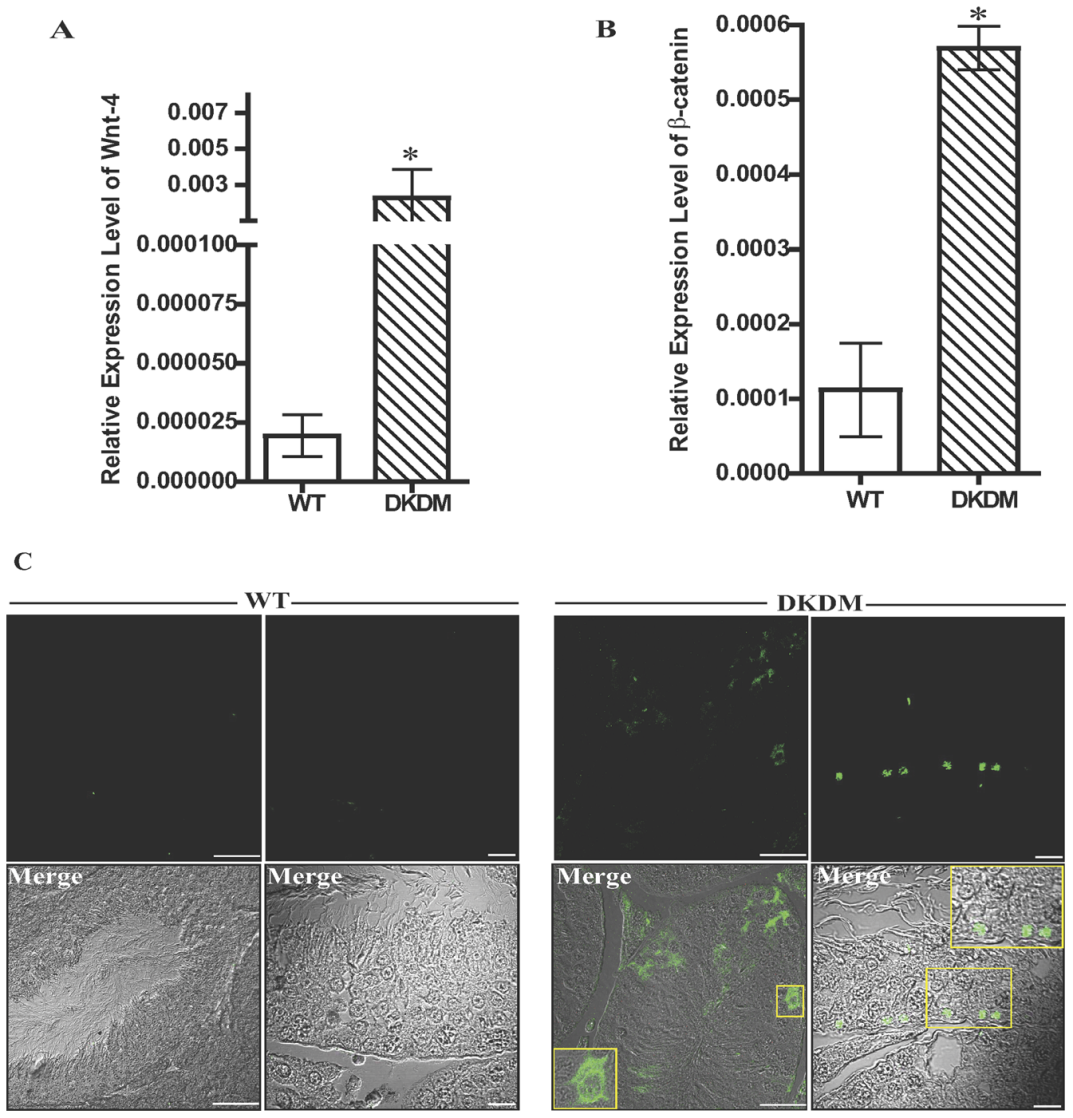
Regulation of WNT signaling by *DKK3*. (A) Relative quantity of *WNT-4* mRNA levels expressed in the testes of WT mice (open bar) and individual DKDM (hatched bar) at ten weeks of age. Real time PCR data from the testicular samples of three animals are represented as mean +/− SEM in each bar. (*p<0.05). (B) Relative quantity of *β-CATENIN* mRNA levels expressed in the testes of WT mice (open bar) and DKDM (hatched bar) at ten weeks of age. Real time PCR data from the testicular samples of three animals are represented as mean +/− SEM in each bar (*p<0.05). (C) Immunohistochemical localization of β-CATENIN in the testicular sections of WT mice and DKDM showing various florescent and merged images. Nuclear localization of β-CATENIN in Sc of DKDM can be seen. Inset in the merged images of DKDM shows magnified area of the boxed regions. All these images are representatives of atleast three random visual fields obtained from atleast three or more animals of each group (WT and DKDM). Left panel florescent and merged images of WT and DKDM, Scale bar: 50 µm. Right panel florescent and merged images of WT and DKDM, Scale bar: 20 µm.

**Figure 6:**
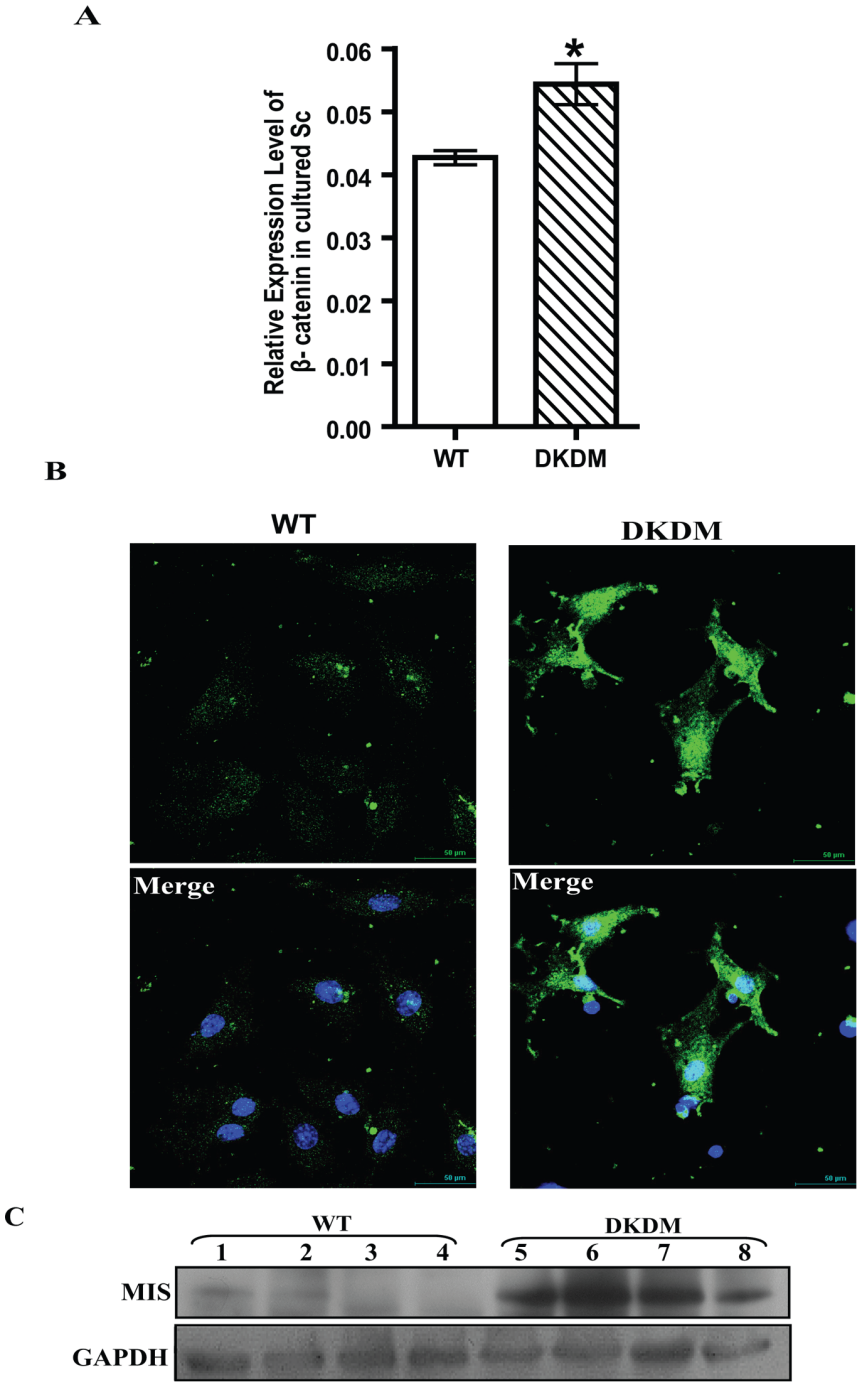
DKK3 mediated regulation of β-CATENIN activity in Sc. (A) Relative quantity of *β-CATENIN* mRNA levels expressed in cultured Sc isolated from WT mice (open bar) and DKDM (hatched bar). Real time PCR data of Sc isolated from four or more individual mice of each group is represented as mean +/− SEM (*p<0.05). (B) Nuclear localization of β-CATENIN in the purified cultures of Sc isolated from WT mice and DKDM. Nuclear localization of β-CATENIN in Sc of DKDM can be seen. Merged image represents nuclear localization of β-CATENIN (green) along with nuclear staining with DAPI (blue). All these images are representatives of atleast three random visual fields obtained from the Sc isolated from four or more individual mice of each group. Scale bar: 50 µm. (C) Western blot analysis of MIS from the testes of WT mice and DKDM at ten weeks of age. Lanes 1–4 represents testicular lysates from four different WT mice, Lanes 5–8 represents testicular lysates from four different DKDM. The quantity of Glyceraldehyde-3-phosphate dehydrogenase (GAPDH) used as a housekeeping gene is shown in the lower panel. Note: equal amount of protein was loaded in each well.

To study the effect of *DKK3* knock down on WNT signaling, the expression of various canonical and non-canonical *WNTs* was analyzed by real-time PCR in DKDM and age matched WT. The expression of WNT-4 was found to be augmented in majority of adult DKDM testis (Fig. 5A). The expression of the other non-canonical WNT mRNAs, including WNT-6, WNT-11, WNT-5a, WNT-5b and canonical WNT-1, WNT-8a did not vary. WNT-3a was not detected at all in the testis of DKDM. Since stabilization of β-CATENIN levels is a known hallmark of activated WNT signaling, we analyzed β-CATENIN expression levels in the testis of DKDM. β-CATENIN mRNA levels were found to be higher in DKDM, sometimes five fold as compared to WT controls (Fig. 5B). Nuclear localization of β-CATENIN was observed in the testicular sections of DKDM in comparison to WT where no nuclear localization of β-CATENIN was observed (Fig. 5C). Similarly, higher expression of β-CATENIN (Fig. 6A) and its nuclear localization was also observed in cultured purified Sc of DKDM (Fig. 6B).

### Suboptimal DKK3 leads to impaired maturation of Sc

MIS expression is a marker of immature Sc and is transcriptionally regulated by β-CATENIN [7]. Therefore, elevated expression of β-CATENIN in adult DKDM prompted us to evaluate the expression of MIS. MIS expression in DKDM was found to remain elevated during adulthood as revealed by real time PCR (Fig. S3A) and western blot analysis (Fig. 6C). Collectively, these observations indicated that in adult DKDM, Sc exhibited gene expression pattern characteristics of undifferentiated Sc, usually found in infant testis.

## Discussion

Our study suggested that augmentation of testicular DKK3 expression observed during pubertal development is crucial for the age dependent onset of spermatogenesis because RNA mediated selective interference in expression of DKK3 interrupted Gc development and fertility. Due to limitations in obtaining normal testes of man, we used Sc from rhesus monkey, a primate known to have close similarity to man. Since it is difficult to pinpoint puberty in man and male monkeys, we generated pubertal monkeys by successful augmentation of hormones and testicular size of juvenile rhesus monkeys upon 4–5 weeks of pulsatile GnRH treatment as previously shown by us [17], [27] and others [28]. Sc of such pubertal monkeys, in which Gc differentiation was markedly initiated, as revealed by presence of spermatocytes, were considered functionally active Sc and used for comparison with Sc obtained from 3 months old infants which were resistant to hormonal stimulation (from 0–3 months) as far as their capacity to induce prolific Gc differentiation was concerned [28]. Rationale to use isolated and cultured Sc instead of whole testicular extract for differential display studies was to determine differential expression of genes exclusively by the Sc which are known to regulate Gc development and differentiation. Since freshly isolated Sc are exposed to repeated enzyme treatments and temperature variations (4°–34°C) during isolation, we preferred to culture and stabilize these cells before evaluating gene expression [6], [29]–[31].

Differential display analysis revealed that *DKK3* expression was significantly elevated during puberty in the Sc of testis engaged in active spermatogenesis as compared to that in Sc during infancy where testis is spermatogenically quiescent, despite of adequate hormones. Dickkopf family of genes comprise of evolutionary conserved four members (*DKK 1*–*4*) and a *DKKL1* or *soggy*. In the past, it had been suggested that *DKK3* does not affect WNT signaling, unlike *DKK 1, DKK 2* and *DKK 4* [11]. However, recently *DKK3* is shown to inhibit canonical WNT signaling specifically in lung and breast cancer cells [15]. Real time PCR analysis in our study, also revealed higher expression of *DKK3* in Sc cultured from testes of pubertal monkeys as compared to those cultured under similar hormonal milieu from the testes of infant monkeys. Since it is difficult to generate transgenic monkeys, we generated *DKK3* knock down mice, referred to as DKDM, to evaluate whether *DKK3* has any regulatory role in spermatogenesis. Before doing so, we confirmed by real time PCR analysis of mouse Sc mRNA that *DKK3* expression was low in 7 days old mice displaying limited Gc differentiation as compared to that in 20 days old mice which displays onset of robust spermatogenesis in the testis.

The DKDM, generated by us showed 40–96% reduction in *DKK3* mRNA expression in testis of mice from F1 generation. Similar suppression of DKK 3 was also observed in cultured Sc of DKDM. Limited number of mice generated using DNA sequences encoding scrambled shRNA did not interfere with *DKK3* expression and their testicular weight, sperm count and fertility were also similar to that of WT mice. “Hence, such mice were not further propagated and WT mice were used instead, for comparison with DKDM”.

DKK3 protein levels were also reduced in DKDM. *DKK3* is a secretory glycoprotein which is found immunohistochemically to be accumulated at Sc-Gc junction of the seminiferous tubules towards lumen [32]. Because it is a secretory protein, it may not be found in abundance in the cytoplasm of Sc. Since we have used CMV promoter, it may express in Gc also [33].

Integration of different copy numbers of the construct (encoding shRNA) in various spermatogonial cells of the testes at the time of gene electroporation might have generated variety of sperm by a single testis. This might be the reason for mice to mice variation in the levels of shRNA expression in the F1 progeny which exerted a broad range of *DKK3* inhibition. Such phenotypic equivalent of a hypomorphic allelic series [34] enabled us to analyze a spectrum of phenotypes ranging from subfertility to infertility in the very first generation of such mice (Supplemental Fig.S2F).

*DKK1, 2, and 4* are known to regulate WNT signaling and bind the same effectors, unlike *DKK3* [11]. However, since DKK3 promoter hyper-methylation mediated decline in DKK3 expression is shown to result into augmentation of WNT signaling in lung cancer cells [15], we evaluated the effect of shRNA mediated DKK3 inhibition on testicular WNT signaling. We found that WNT-4 was highly expressed in adult DKDM testis as compared to age matched WT testis. However, the expression of the other non-canonical WNT mRNAs in DKDM, including WNT-6, WNT-11, WNT-5a, WNT-5b and canonical WNT-1, WNT-8a did not vary notably as compared to age matched WT mice.

In testes of DKDM, enhanced WNT-4 signaling was associated with an increase in β-CATENIN mRNA levels. Such directly proportional relationship between WNT-4 and β-CATENIN had been shown in the past [35]. It is known that β-CATENIN enhances WNT-4 promoter activity [36] and also activates its own promoter, leading to a further up regulation of β-CATENIN expression [37]. This may be the reason for about five fold or more rise in β-CATENIN m-RNA levels in some of the DKDM. Since nuclear localization of β-CATENIN is indicator of its activity leading to augmentation of transcriptional events, we evaluated and confirmed elevated nuclear localization of β-CATENIN in seminiferous tubular cells and cultured Sc of DKDM in comparison to WT. In testicular section, both Sc and Gc displayed nuclear localization of β-CATENIN because DKK3 has also been shown to be expressed in Gc [33]. The nuclear localization of β-CATENIN upon down regulation of DKK3 in DKDM suggested inhibition of β-CATENIN ubiquitination in DKDM. No specific staining for β-CATENIN was seen in any of the cells of WT mice testis, probably due to its complete degradation by ubiquitination. This suggested that nuclear localization of non-degraded β-CATENIN occurred in testicular cells of mice when *DKK3* was knocked down.

Differentiation of spermatogonial cells is dependent on testicular Sc, which have receptors for both FSH and T [29], [38]. We have previously shown that differentiated, mature Sc alter their pattern of gene expression during puberty which are associated with onset of spermatogenesis [6], [30], [39]. In DKDM, augmentation of WNT-4/β-CATENIN activity due to inhibition of *DKK3* expression might have restricted Sc maturation and therefore impaired spermatogenesis in F1 generation. Support to this observation is lent from a very recent study where augmentation of WNT-4/β-CATENIN signaling resulted in incomplete differentiation of Sc and loss of Gc [35].

Conditionally activated allele of the β-CATENIN in Sc expressing Cre recombinase driven by the MIS promoter has already been shown to constitutively activate β-CATENIN, leading to continuous proliferation and compromised differentiation of mouse Sc [7]. As compared to WT mice, Sc in such adult mutant mice continued to express high levels of MIS which is characteristic of immature Sc. Our observation of elevated MIS expression in DKDM suggested failure of Sc maturation due to diminished DKK3. Additionally, reduced diameter of seminiferous tubule in DKDM which is also one of the determinants of Sc maturation also suggested that Sc were immature in DKDM.

Sc also possess phagocytic activity where Sc recognize apoptotic spermatogenic cells through the binding of their surface receptor, class B scavenger receptor type I, to phosphatidylserine that is expressed on the surface of spermatogenic cells during apoptosis [40]. Inhibition of phagocytosis is known to reduce the number of epididymal sperm, indicating that phagocytosis of apoptotic spermatogenic cells by Sc is necessary for efficient production of sperm. If ability of Sc to phagocytose the apoptotic cells declines while maintaining their ability to support spermatogenesis, accumulation of apoptotic Gc may further affect normal functioning of Sc including sloughing off the Gc, as observed by us in DKDM [40].

High levels of MIS expression in adult testis of DKDM also might have contributed to infertility since MIS over expression is associated with severe testicular defects [41]. MIS is also known to exert a negative effect upon steroidogenesis by decreasing expression of CYP17, an enzyme involved in androgen biosynthesis in testes [42]. In DKDM, enhanced MIS expression was associated with reduction in CYP17 mRNA levels which might have contributed to the reduced T levels. However, this decrease in T levels was not the primary cause of subfertility observed in these mice because hormone replacement by exogenous supplementation of T failed to restore normal spermatogenesis in DKDM. DKK3 is also known to be expressed in pituitary and hypothalamus [43] but in mice with mutated *DKK3*, fertility is not compromised indicating lack of any systemic effect on fertility via pituitary and/or hypothalamus [44]. Also, in DKDM mice generated by us, most of the mice from F1 generation were able to produce sperm as well as progeny, only the number of pups and sperm count were low. This cannot be the effect of systemic changes in hypothalamic or pituitary hormones because in such a situation azoospermia would prevail, which was not the case. In DKDM, levels of testosterone declined but replenishing testosterone by exogenous treatment did not result into restoration of sperm production, making us to believe that the effect was not because of the change in T levels but because of other changes at the level of testis. Although it cannot be ruled out that DKK3 down regulation might have affected Leydig cells (Lc), function of DKK3 is uncertain in Lc [45]. Main contribution of Lc is to provide T to promote spermatogenesis and replenishment of T in our study failed to recover the disturbed spermatogenic status of these transgenic mice, letting us believe that DKK3 mediated effect is non-hormonal and at the level of Sc.

LH and FSH levels were not checked because most of F1 generation mice were fertile and sired live offspring also. This could not have happened if levels of FSH and LH were physiologically below optimal. Additionally, mating of DKDM females with WT males yielded normal litter size suggesting no defect in gonadotropins due to DKK3 inhibition which might have disturbed normal ovulation in DKDM females.

It is reasonable to assume from our study that reduction in DKK3 causes activation of WNT/β-CATENIN activity in testis restricting maturation of Sc which leads to subfertility or infertility. While *DKK3* mutant mice have been generated previously, no long term study of male fertility in them was undertaken as they were initially found to be fertile [44]. Differences in the fertility of *DKK3* knock out and our knock down mice could be explained by the possibility of redundancy in *DKK3* mutant mice where function of *DKK3* might have been taken over by some other genes [46]. However, combined knocking out of *DKK3* and the unique *DKK3*-*homolog, SOGGY* [13] may reveal roles masked by redundancy. However, since shRNA mediated interference occurs in the cytoplasm, such redundancy is not possible in knock down mice.

We used *CMV* promoter because it is a known strong promoter which guaranteed the expression of shRNA *in vivo* [47]. Therefore, we evaluated functional aspects of gene using ubiquitous promoter like CMV [48], [49] for our DKK3 shRNA knock down study knowing that shRNA is expected to express ubiquitously in other tissues also to bring down DKK3 expression, if any. In knockout animals which successfully divulged roles of several genes, the expression of the genes are also lost ubiquitously [44]. However, studies of Sc specific promoter mediated shRNA expression to inhibit *DKK3* exclusively in Sc, *may* further strengthen our findings.

**Figure 7:**
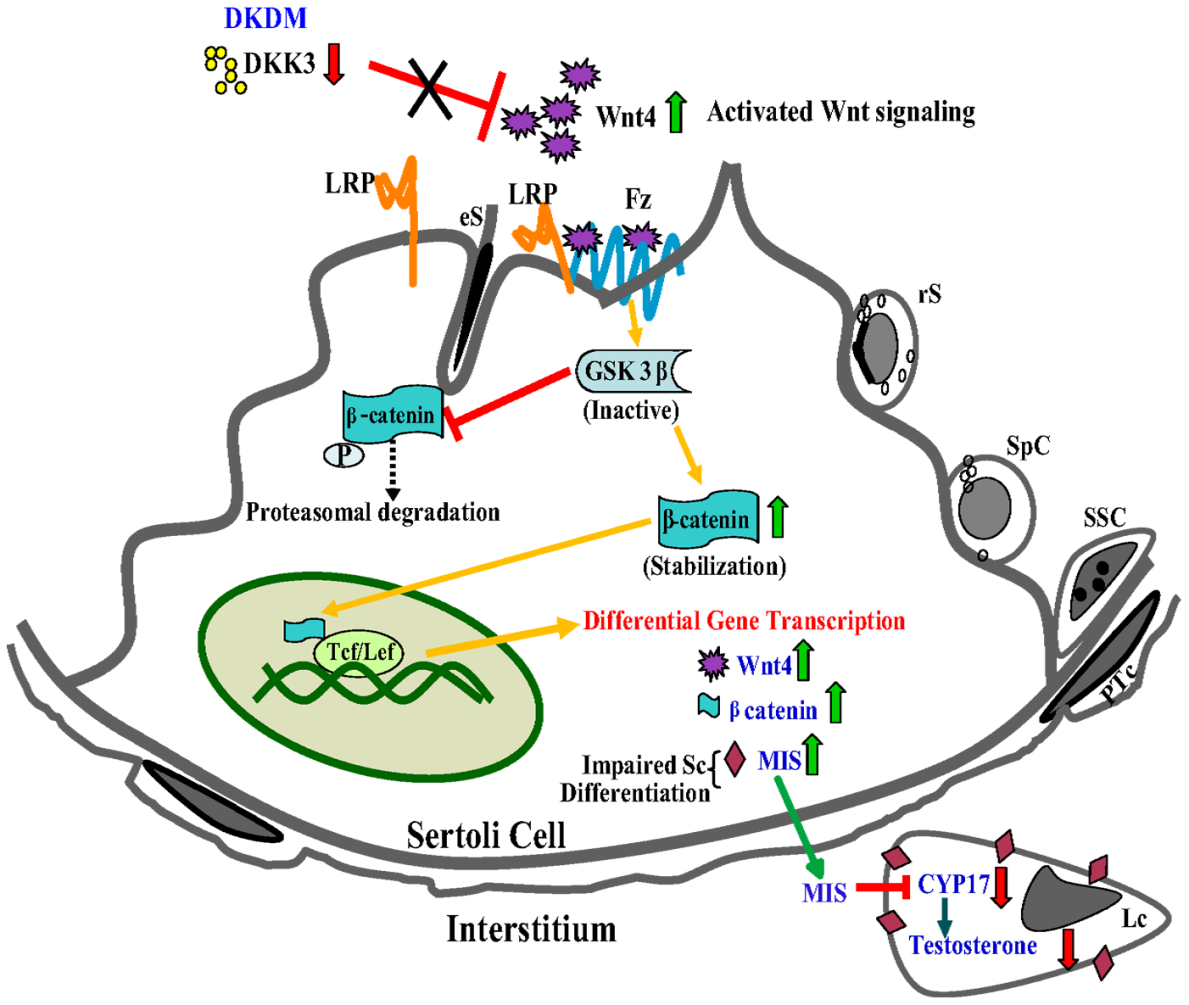
A model of DKK3 mediated regulation of Sc maturation and spermatogenesis. In DKDM, LRP6 becomes available because of diminished *DKK3* levels. Available *LRP6* binds to Frizzled receptor forming *Fz-*LRP6 complex augmenting WNT signaling. As a result, *GSK-3β* is recruited to Frizzled receptor reducing its availability for binding to β-CATENIN, thereby minimizing phosphorylation and degradation of β-CATENIN. Accumulated cytoplasmic β-CATENIN translocates to nucleus and augments expression of different genes, including that of itself, WNT4 and MIS in Sc. Elevated levels of MIS interferes with the Sc maturation, hence, disturbing the balance between spermatogonial proliferation and differentiation leading to subfertility and/or infertility. Additionally, *MIS* is known to inhibit T production via suppression of CYP17 activity in Lc which might be the reason for reduced T levels in such mice. (eS-elongated spermatids, SSC-spermatogonial stem cells, Fz-Frizzled, rS-round spermatid, SpC-spermatocytes, PTc-peritubular cell, Tcf/Lcf -transcription factors).

In a recent study, it has been demonstrated that WNT/β-CATENIN signaling pathway influences spermatogenesis [7], [35]. Our study further confirmed these findings. This study provided substantial support to the notion that a remarkable rise observed in DKK3 expression during puberty, acts as a suppressor of WNT-4 signaling allowing maturation of Sc during natural course of development (Fig.7). A rise in WNT-4 activity associated with a corresponding rise in β-CATENIN activity as a result of diminished DKK3 expression in DKDM was responsible for impaired Sc differentiation.

In conclusion, we have found a novel role for *DKK3* upstream of WNT-4 signaling in Sc, positioning *DKK3* as a key regulator of Sc maturation which is an essential prerequisite for normal spermatogenesis observed during puberty (Fig.S4). Disruption of DKK3 expression in Sc due to natural mutation or due to induced effect (environmental or other causes) may interfere with Sc maturation leading to subfertility or infertility even if hormones and their receptors are normal. These findings highlighted the possibility of new therapeutical strategies for overcoming similar idiopathic infertility observed in humans and suggested that restoration of fertility can be achieved by correction certain genes raising hopes for individuals suffering from idiopathic male infertility.

## Supporting Information

Figure S1 (A) Fluorescent Differential display using mRNA from hormone (FSH and T) treated Sc cultured from testis of infant (In) and pubertal (Pu) monkey. For each set of arbitrary primers, 4 lanes were loaded with PCR product (Lane 1 and 2 are replicates of infant monkey samples, lane 3 and 4 are replicates of pubertal monkey samples). Arrow shows higher expression of sequences specific to *DKK3* in pubertal Sc as compared to infant Sc. (B) A cartoon of the construct used for the generation of DKDM. A linearized shRNA vector consisting of *CMV* promoter which drives the expression of sequence for *DKK3* shRNA and a SV40 promoter which drives the expression of GFP. Gene specific shRNA sequences were inserted into the vector in between BamH1 and AflII sites. (C) Immunohistochemical localization of GFP in the testicular sections showing fluorescence and merged images of WT mice (no GFP expression) and DKDM (expressing GFP) at ten weeks of age, Scale bar: 50 µm. All these images are representatives of atleast three random visual fields obtained from atleast three or more animals of each group (WT and DKDM).
(TIF)

Figure S2 (A) Mean testis weight (in mgs) of WT mice (open bar) and control mice generated using scrambled *DKK3 shRNA* construct (hatched bar) at ten weeks of age. There was no statistical difference between two groups (n = 10, p<0.05). (B) Mean epididymal sperm counts (million/ml) of WT mice (open bar) and control mice generated using scrambled *DKK3 shRNA* construct (hatched bar) at ten weeks of age. There was no statistical difference between two groups (p<0.05, n = 10). (C) Mean litter size of WT mice (open bar) and control mice generated using scrambled *DKK3 shRNA* construct (hatched bar) at ten weeks of age. There was no statistical difference between two groups (p<0.05, n = 3). (D) Serum Testosterone levels of WT mice (open bar) and control mice generated using scrambled *DKK3 shRNA* construct (hatched bar) at ten weeks of age. There was no statistical difference between two groups (p<0.05, n = 3). (E) Mean body weight of WT mice (open bar) and DKDM (hatched bar) at ten weeks of age. Data are represented as mean +/− SEM (*p<0.05, n = 10). (F) Mean testis weight of WT mice (open bar) and DKDM (hatched bar) at ten weeks of age. Data are represented as mean +/− SEM (*p<0.05, n = 10). (G) Real time PCR showing relative fold expression of *DKK3 in* WT mice and DKDM mice (open bar, left side Y-axis). D1-D3 represents testicular samples from three individual DKDM. Hatched bar (towards right side Y-axis) shows epididymal sperm counts from the same three DKDM mice depicting spectrum of phenotypes ranging from oligospermia to azoospermia in F1 generation. Mean value of *DKK3* expression in WT mice (n = 3) were considered as 1 for comparison. (H) TUNEL assay detecting apoptotic cells in the testis of WT mice and DKDM at ten weeks of age, Scale bar 50 µm. Higher number of apoptotic cells were seen in the testicular sections of DKDM as compared to WT controls. All these images are representatives of atleast three random visual fields obtained from atleast three or more animals of each group (WT and DKDM).
(TIF)

Figure S3 (A) Relative expression levels of *MIS* in the testes of WT mice (open bar) and individual DKDM (hatched bars) at ten weeks of age. Real time PCR data from the testicular samples of three animals are represented as mean +/− SEM in each bar (*p<0.05). (B) Mean litter size from the matings of WT males with WT females (open bar), DKDM males with WT females (dotted bar) and DKDM females with WT males (hatched bar) at ten weeks of age (*p<0.05, n = 3).
(TIF)

Figure S4 **A cartoon showing that upregulation of DKK3 expression during natural course to puberty (yellow section) is responsible for Sc maturation and that inhibition of DKK3 in transgenic DKDM results into maturational failure of Sc (dark pink section).**
(TIF)

Table S1 **List of primers used for Real time PCR.**
(DOC)

## References

[1] Matzuk MM, Lamb DJ (2008) The biology of infertility: research advances and clinical challenges. Nat Med 14: 1197–1213. 18989307 10.1038/nm.f.1895 PMC3786590

[2] Ikawa M, Tergaonkar V, Ogura A, Ogonuki N, Inoue K, et al (2002) Restoration of spermatogenesis by lentiviral gene transfer: offspring from infertile mice. Proc Natl Acad Sci USA 99: 7524–7529. 12032316 10.1073/pnas.072207299 PMC124271

[3] Schaison G, Young J, Pholsena M, Nahoul K, Couzinet B (1993) Failure of combined follicle-stimulating hormone-testosterone administration to initiate and/or maintain spermatogenesis in men with hypogonadotropic hypogonadism. J Clin Endocrinol Metab 77: 1545–1549. 8263139 10.1210/jcem.77.6.8263139

[4] Walker WH, Cheng J (2005) FSH and testosterone signaling in Sertoli cells. Reproduction 130: 15–28. 15985628 10.1530/rep.1.00358

[5] Majumdar SS, Bhattacharya I (2012) Genomic and post-genomic leads toward regulation of spermatogenesis. Prog Biophys Mol Biol 27: 2515–2525. 10.1016/j.pbiomolbio.2013.01.002 23375691

[6] Bhattacharya I, Pradhan BS, Sarda K, Gautam M, Basu S, et al (2012) A switch in Sertoli cell responsiveness to FSH may be responsible for robust onset of germ cell differentiation during prepubartal testicular maturation in rats. Am J Physiol Endocrinol Metab 303: E886–898. 22850685 10.1152/ajpendo.00293.2012

[7] Tanwar PS, Kaneko-Tarui T, Zhang L, Rani P, Taketo MM, et al (2010) Constitutive WNT/beta-catenin signaling in murine Sertoli cells disrupts their differentiation and ability to support spermatogenesis. Biol Reprod 82: 422–432. 19794154 10.1095/biolreprod.109.079335 PMC2809230

[8] Golestaneh N, Beauchamp E, Fallen S, Kokkinaki M, Uren A, et al (2009) Wnt signaling promotes proliferation and stemness regulation of spermatogonial stem/progenitor cells. Reproduction 138: 151–162. 19419993 10.1530/REP-08-0510

[9] Boyer A, Hermo L, Paquet M, Robaire B, Boerboom D (2008) Seminiferous tubule degeneration and infertility in mice with sustained activation of WNT/CTNNB1 signaling in sertoli cells. Biol Reprod 79: 475–485. 18480464 10.1095/biolreprod.108.068627

[10] Meng X, Lindahl M, Hyvonen ME, Parvinen M, de Rooij DG, et al (2000) Regulation of cell fate decision of undifferentiated spermatogonia by GDNF. Science 287: 1489–1493. 10688798 10.1126/science.287.5457.1489

[11] Niehrs C (2006) Function and biological roles of the Dickkopf family of Wnt modulators. Oncogene 25: 7469–7481. 17143291 10.1038/sj.onc.1210054

[12] Glinka A, Wu W, Delius H, Monaghan AP, Blumenstock C, et al (1998) Dickkopf-1 is a member of a new family of secreted proteins and functions in head induction. Nature 391: 357–362. 9450748 10.1038/34848

[13] Krupnik VE, Sharp JD, Jiang C, Robison K, Chickering TW, et al (1999) Functional and structural diversity of the human Dickkopf gene family. Gene 238: 301–313. 10570958 10.1016/s0378-1119(99)00365-0

[14] Mao B, Wu W, Li Y, Hoppe D, Stannek P, et al (2001) LDL-receptor-related protein 6 is a receptor for Dickkopf proteins. Nature 411: 321–325. 11357136 10.1038/35077108

[15] Lee EJ, Jo M, Rho SB, Park K, Yoo YN, et al (2009) Dkk3, downregulated in cervical cancer, functions as a negative regulator of beta-catenin. Int J Cancer 124: 287–297. 19003969 10.1002/ijc.23913

[16] Veeck J, Dahl E (2012) Targeting the Wnt pathway in cancer: The emerging role of Dickkopf-3. Biochim Biophys Acta 1825: 18–28. 21982838 10.1016/j.bbcan.2011.09.003

[17] Devi YS, Sarda K, Stephen B, Nagarajan P, Majumdar SS (2006) Follicle-stimulating hormone-independent functions of primate Sertoli cells: potential implications in the diagnosis and management of male infertility. J Clin Endocrinol Metab 91: 1062–1068. 16368746 10.1210/jc.2005-2072

[18] Majumdar SS, Winters SJ, Plant TM (1998) Procedures for the isolation and culture of Sertoli cells from the testes of infant, juvenile, and adult rhesus monkeys (Macaca mulatta). Biol Reprod 58: 633–640. 9510950 10.1095/biolreprod58.3.633

[19] Welsh MJ, Wiebe JP (1975) Rat sertoli cells: a rapid method for obtaining viable cells. Endocrinology 96: 618–624. 163729 10.1210/endo-96-3-618

[20] Liang P, Pardee AB (1992) Differential display of eukaryotic messenger RNA by means of the polymerase chain reaction. Science 257: 967–971. 1354393 10.1126/science.1354393

[21] Dhup S, Majumdar SS (2008) Transgenesis via permanent integration of genes in repopulating spermatogonial cells in vivo. Nat Methods 5: 601–603. 18552853 10.1038/nmeth.1225

[22] Hogan B, Beddington R, Constantini F, Lacy E (1994) Manipulating the mouse Embryo: A Laboratory Manual.New York:Cold Spring Harbor press. 180 p.

[23] Schmittgen TD, Livak KJ (2008) Analyzing real-time PCR data by the comparative C(T) method. Nat Protoc 3: 1101–1108. 18546601 10.1038/nprot.2008.73

[24] Lo KC, Ivanova M, Oesterreich S, Lamb DJ (2004) Androgen replacement restores spermatogenesis in SAFB1 knockout mice. Fertility and sterility 82: S22–S23.15363688

[25] Laemmli UK (1970) Cleavage of structural proteins during the assembly of the head of bacteriophage T4. Nature 227: 680–685. 5432063 10.1038/227680a0

[26] Plant TM, Witchel SF(2006) Puberty in non-human primates and humans. In: Challis JRG, Neill JD, Pfaff DW, Plant TM, Richards JS, et al.., editors. Knobil and Neill's Physiology of Reproduction. 3^rd^ edition, Vol. 2 San Diego: USA.pp. 2177–2230.

[27] Majumdar SS, Mikuma N, Ishwad PC, Winters SJ, Attardi BJ, et al (1995) Replacement with recombinant human inhibin immediately after orchidectomy in the hypophysiotropically clamped male rhesus monkey (*Macaca mulatta*) maintains follicle-stimulating hormone (FSH) secretion and FSH beta messenger ribonucleic acid levels at precastration values. Endocrinology 136: 1969–1977. 7720645 10.1210/endo.136.5.7720645

[28] Plant TM, Ramaswamy S, Simorangkir D, Marshall GR (2005) Postnatal and pubertal development of the rhesus monkey (Macaca mulatta) testis. Ann N Y Acad Sci 1061: 149–162. 16467264 10.1196/annals.1336.016

[29] Griswold MD (1998) The central role of Sertoli cells in spermatogenesis. Semin Cell Dev Biol 9: 411–416. 9813187 10.1006/scdb.1998.0203

[30] Majumdar SS, Sarda K, Bhattacharya I, Plant TM (2012) Insufficient androgen and FSH signaling may be responsible for the azoospermia of the infantile primate testes despite exposure to an adult-like hormonal milieu. Hum Reprod 27: 2515–2525. 22669085 10.1093/humrep/des184 PMC3398678

[31] Skinner MK, Griswold MD (1982) Secretion of testicular transferrin by cultured Sertoli cells is regulated by hormones and retinoids. Biol Reprod 27: 211–221. 6810965 10.1095/biolreprod27.1.211

[32] Grima J, Zhu L, Cheng CY (1997) Testin is tightly associated with testicular cell membrane upon its secretion by sertoli cells whose steady-state mRNA level in the testis correlates with the turnover and integrity of inter-testicular cell junctions. J Biol Chem 272: 6499–6509. 9045675 10.1074/jbc.272.10.6499

[33] Tanimoto R, Abarzua F, Sakaguchi M, Takaishi M, Nasu Y, et al (2007) REIC/Dkk-3 as a potential gene therapeutic agent against human testicular cancer. Int J Mol Med 19: 363–368. 17273781

[34] Kunath T, Gish G, Lickert H, Jones N, Pawson T, et al (2003) Transgenic RNA interference in ES cell-derived embryos recapitulates a genetic null phenotype. Nat Biotechnol 21: 559–561. 12679785 10.1038/nbt813

[35] Boyer A, Yeh JR, Zhang X, Paquet M, Gaudin A, et al (2012) CTNNB1 signaling in sertoli cells downregulates spermatogonial stem cell activity via WNT4. PLoS One 7: e29764. 22253774 10.1371/journal.pone.0029764 PMC3257228

[36] Chang H, Gao F, Guillou F, Taketo MM, Huff V, et al (2008) Wt1 negatively regulates beta-catenin signaling during testis development. Development 135: 1875–1885. 18403409 10.1242/dev.018572 PMC4038296

[37] Bandapalli OR, Dihlmann S, Helwa R, Macher-Goeppinger S, Weitz J, et al (2009) Transcriptional activation of the beta-catenin gene at the invasion front of colorectal liver metastases. J Pathol 218: 370–379. 19347947 10.1002/path.2539

[38] Orth JM, Gunsalus GL, Lamperti AA (1988) Evidence from Sertoli cell-depleted rats indicates that spermatid number in adults depends on numbers of Sertoli cells produced during perinatal development. Endocrinology 122: 787–794. 3125042 10.1210/endo-122-3-787

[39] Sharpe RM, McKinnell C, Kivlin C, Fisher JS (2003) Proliferation and functional maturation of Sertoli cells, and their relevance to disorders of testis function in adulthood. Reproduction 125: 769–784. 12773099 10.1530/rep.0.1250769

[40] Nakanishi Y, Shiratsuchi A (2004) Phagocytic removal of apoptotic spermatogenic cells by Sertoli cells: mechanisms and consequences. Biol Pharm Bull 27: 13–16. 14709891 10.1248/bpb.27.13

[41] Behringer RR, Cate RL, Froelick GJ, Palmiter RD, Brinster RL (1990) Abnormal sexual development in transgenic mice chronically expressing mullerian inhibiting substance. Nature 345: 167–170. 2336108 10.1038/345167a0

[42] Racine C, Rey R, Forest MG, Louis F, Ferre A, et al (1998) Receptors for anti-mullerian hormone on Leydig cells are responsible for its effects on steroidogenesis and cell differentiation. Proc Natl Acad Sci U S A 95: 594–599. 9435237 10.1073/pnas.95.2.594 PMC18465

[43] Diep DB, Hoen N, Backman M, Machon O, Krauss S (2004) Characterisation of the Wnt antagonists and their response to conditionally activated Wnt signalling in the developing mouse forebrain. Brain Res Dev Brain Res 153: 261–270. 15527894 10.1016/j.devbrainres.2004.09.008

[44] Barrantes Idel B, Montero-Pedrazuela A, Guadano-Ferraz A, Obregon MJ, Martinez de Mena R, et al (2006) Generation and characterization of dickkopf3 mutant mice. Mol Cell Biol 26: 2317–2326. 16508007 10.1128/MCB.26.6.2317-2326.2006 PMC1430294

[45] Abel MH, Baban D, Lee S, Charlton HM, O′Shaughnessy PJ (2009) Effects of FSH on testicular mRNA transcript levels in the hypogonadal mouse. J Mol Endocrinol 42: 291–303. 19136570 10.1677/JME-08-0107 PMC2659293

[46] Gossen M, Bujard H (2002) Studying gene function in eukaryotes by conditional gene inactivation. Annu Rev Genet 36: 153–173. 12429690 10.1146/annurev.genet.36.041002.120114

[47] Xia H, Mao Q, Paulson HL, Davidson BL (2002) siRNA-mediated gene silencing in vitro and in vivo. Nat Biotechnol 20: 1006–1010. 12244328 10.1038/nbt739

[48] Muller SR, Sullivan PD, Clegg DO, Feinstein SC (1990) Efficient transfection and expression of heterologous genes in PC12 cells. DNA Cell Biol 9: 221–229. 2187480 10.1089/dna.1990.9.221

[49] Okabe S, Forsberg-Nilsson K, Spiro AC, Segal M, McKay RD (1996) Development of neuronal precursor cells and functional postmitotic neurons from embryonic stem cells in vitro. Mech Dev 59: 89–102. 8892235 10.1016/0925-4773(96)00572-2

