# Correction: Dickkopf Homolog 3 (*DKK3*) Plays a Crucial Role Upstream of WNT/β-CATENIN Signaling for Sertoli Cell Mediated Regulation of Spermatogenesis

**DOI:** 10.1371/annotation/a05eeef0-0251-4c4a-bed6-45b022797c74

**Published:** 2014-01-21

**Authors:** Deepika Sharma Das, Neerja Wadhwa, Neetu Kunj, Kanchan Sarda, Bhola Shankar Pradhan, Subeer S. Majumdar

In the Reference section, Reference 5 and 6 should be replaced with the following:

5. Bhattacharya I, Pradhan BS, Sarda K, Gautam M, Basu S, et al. (2012) A switch in Sertoli cell responsiveness to FSH may be responsible for robust onset of germ cell differentiation during prepubartal testicular maturation in rats. Am J Physiol Endocrinol Metab 303: E886–898. http://dx.doi.org/10.1152/ajpendo.00293.2012

6. Majumdar SS, Sarda K, Bhattacharya I, Plant TM (2012) Insufficient androgen and FSH signaling may be responsible for the azoospermia of the infantile primate testes despite exposure to an adult-like hormonal milieu. Hum. Reprod. 27 : 2515–2525 

