# Supplementary figures and images for: Dickkopf Homolog 3 (DKK3) Plays a Crucial Role Upstream of WNT/β-CATENIN Signaling for Sertoli Cell Mediated Regulation of Spermatogenesis
Source: PLoS One. 2013 May 7;8(5):e63603. doi: 10.1371/journal.pone.0063603 (PMC3647036; doi:10.1371/journal.pone.0063603)

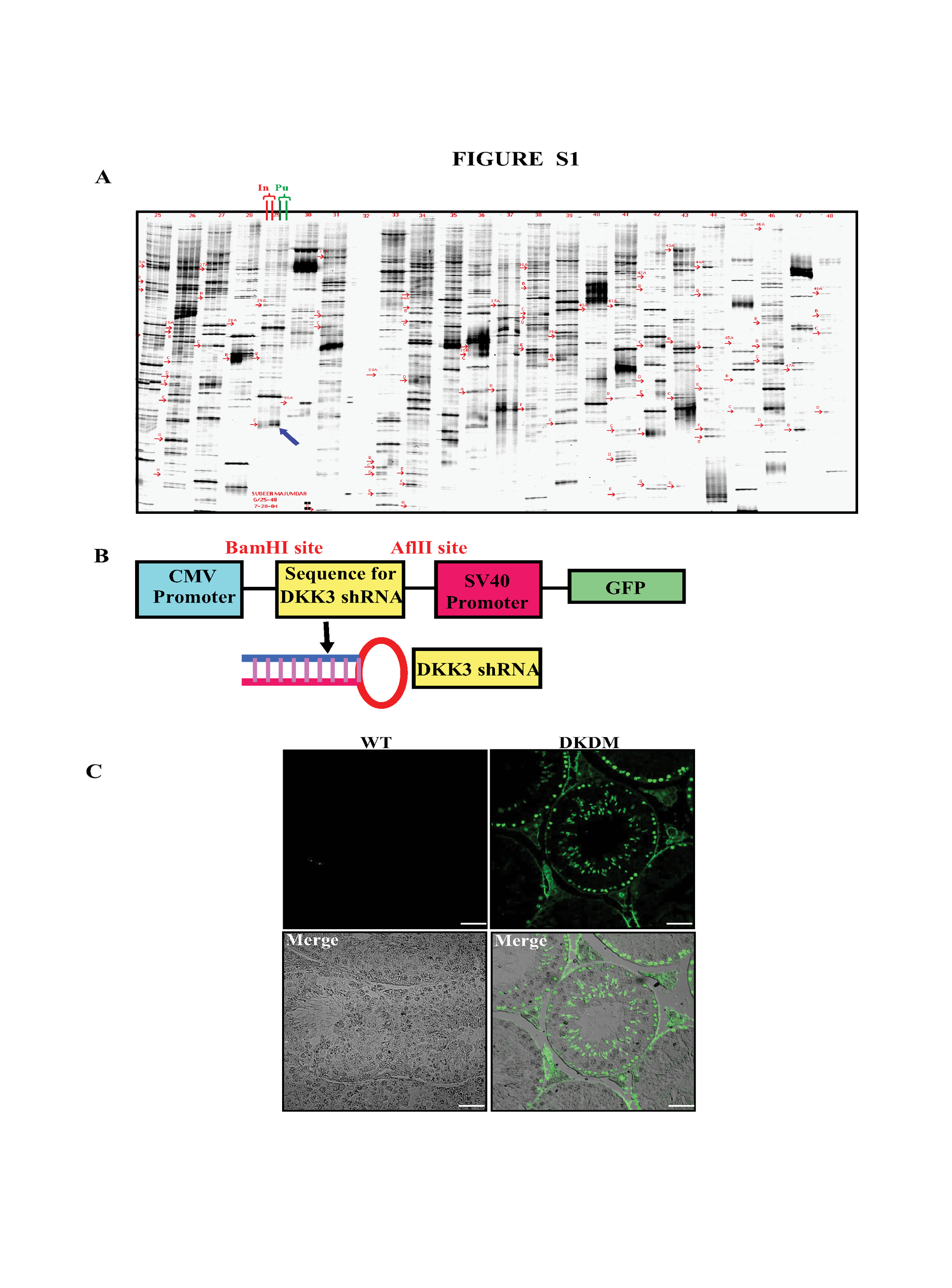

Supplement: Figure S1 — (A) Fluorescent Differential display using mRNA from hormone (FSH and T) treated Sc cultured from testis of infant (In) and pubertal (Pu) monkey. For each set of arbitrary primers, 4 lanes were loaded with PCR product (Lane 1 and 2 are replicates of infant monkey samples, lane 3 and 4 are replicates of pubertal monkey samples). Arrow shows higher expression of sequences specific to DKK3 in pubertal Sc as compared to infant Sc. (B) A cartoon of the construct used for the generation of DKDM. A linearized shRNA vector consisting of CMV promoter which drives the expression of sequence for DKK3 shRNA and a SV40 promoter which drives the expression of GFP. Gene specific shRNA sequences were inserted into the vector in between BamH1 and AflII sites. (C) Immunohistochemical localization of GFP in the testicular sections showing fluorescence and merged images of WT mice (no GFP expression) and DKDM (expressing GFP) at ten weeks of age, Scale bar: 50 µm. All these images are representatives of atleast three random visual fields obtained from atleast three or more animals of each group (WT and DKDM). (TIF) [file pone.0063603.s001.tif]

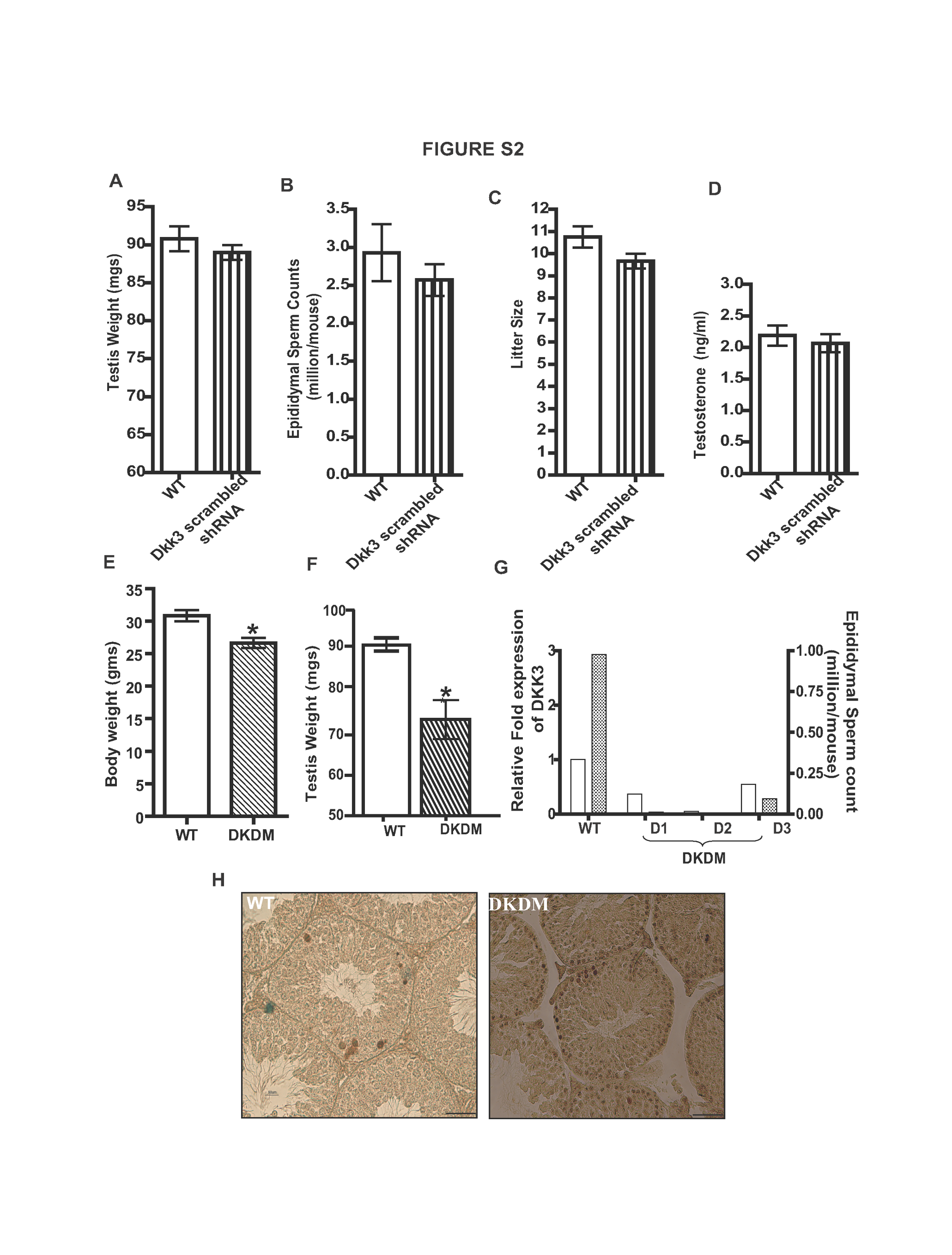

Supplement: Figure S2 — (A) Mean testis weight (in mgs) of WT mice (open bar) and control mice generated using scrambled DKK3 shRNA construct (hatched bar) at ten weeks of age. There was no statistical difference between two groups (n = 10, p<0.05). (B) Mean epididymal sperm counts (million/ml) of WT mice (open bar) and control mice generated using scrambled DKK3 shRNA construct (hatched bar) at ten weeks of age. There was no statistical difference between two groups (p<0.05, n = 10). (C) Mean litter size of WT mice (open bar) and control mice generated using scrambled DKK3 shRNA construct (hatched bar) at ten weeks of age. There was no statistical difference between two groups (p<0.05, n = 3). (D) Serum Testosterone levels of WT mice (open bar) and control mice generated using scrambled DKK3 shRNA construct (hatched bar) at ten weeks of age. There was no statistical difference between two groups (p<0.05, n = 3). (E) Mean body weight of WT mice (open bar) and DKDM (hatched bar) at ten weeks of age. Data are represented as mean +/− SEM (*p<0.05, n = 10). (F) Mean testis weight of WT mice (open bar) and DKDM (hatched bar) at ten weeks of age. Data are represented as mean +/− SEM (*p<0.05, n = 10). (G) Real time PCR showing relative fold expression of DKK3 in WT mice and DKDM mice (open bar, left side Y-axis). D1-D3 represents testicular samples from three individual DKDM. Hatched bar (towards right side Y-axis) shows epididymal sperm counts from the same three DKDM mice depicting spectrum of phenotypes ranging from oligospermia to azoospermia in F1 generation. Mean value of DKK3 expression in WT mice (n = 3) were considered as 1 for comparison. (H) TUNEL assay detecting apoptotic cells in the testis of WT mice and DKDM at ten weeks of age, Scale bar 50 µm. Higher number of apoptotic cells were seen in the testicular sections of DKDM as compared to WT controls. All these images are representatives of atleast three random visual fields obtained from atleast three or more animals of each group [file pone.0063603.s002.tif]

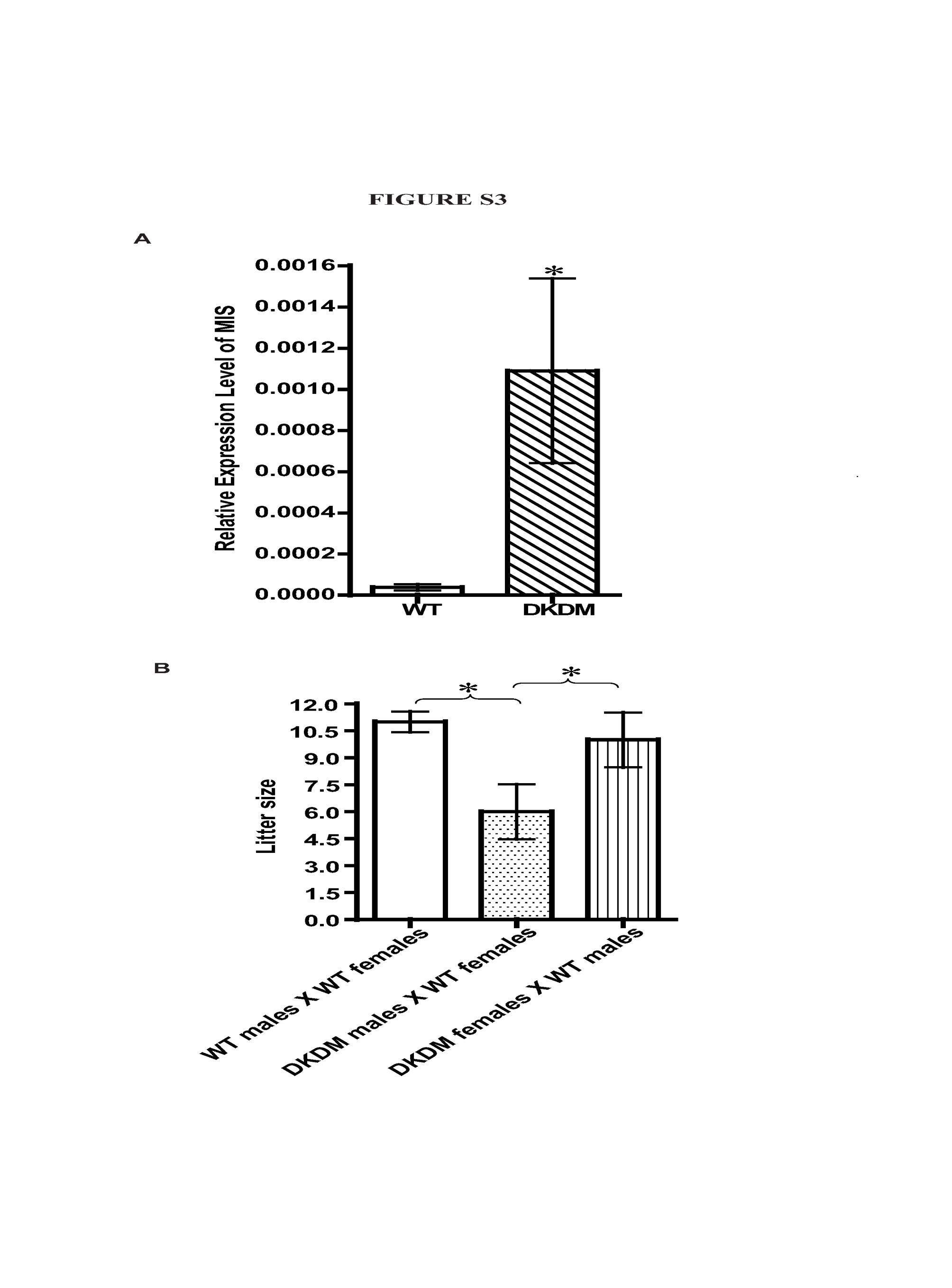

Supplement: Figure S3 — (A) Relative expression levels of MIS in the testes of WT mice (open bar) and individual DKDM (hatched bars) at ten weeks of age. Real time PCR data from the testicular samples of three animals are represented as mean +/− SEM in each bar (*p<0.05). (B) Mean litter size from the matings of WT males with WT females (open bar), DKDM males with WT females (dotted bar) and DKDM females with WT males (hatched bar) at ten weeks of age (*p<0.05, n = 3). (TIF) [file pone.0063603.s003.tif]

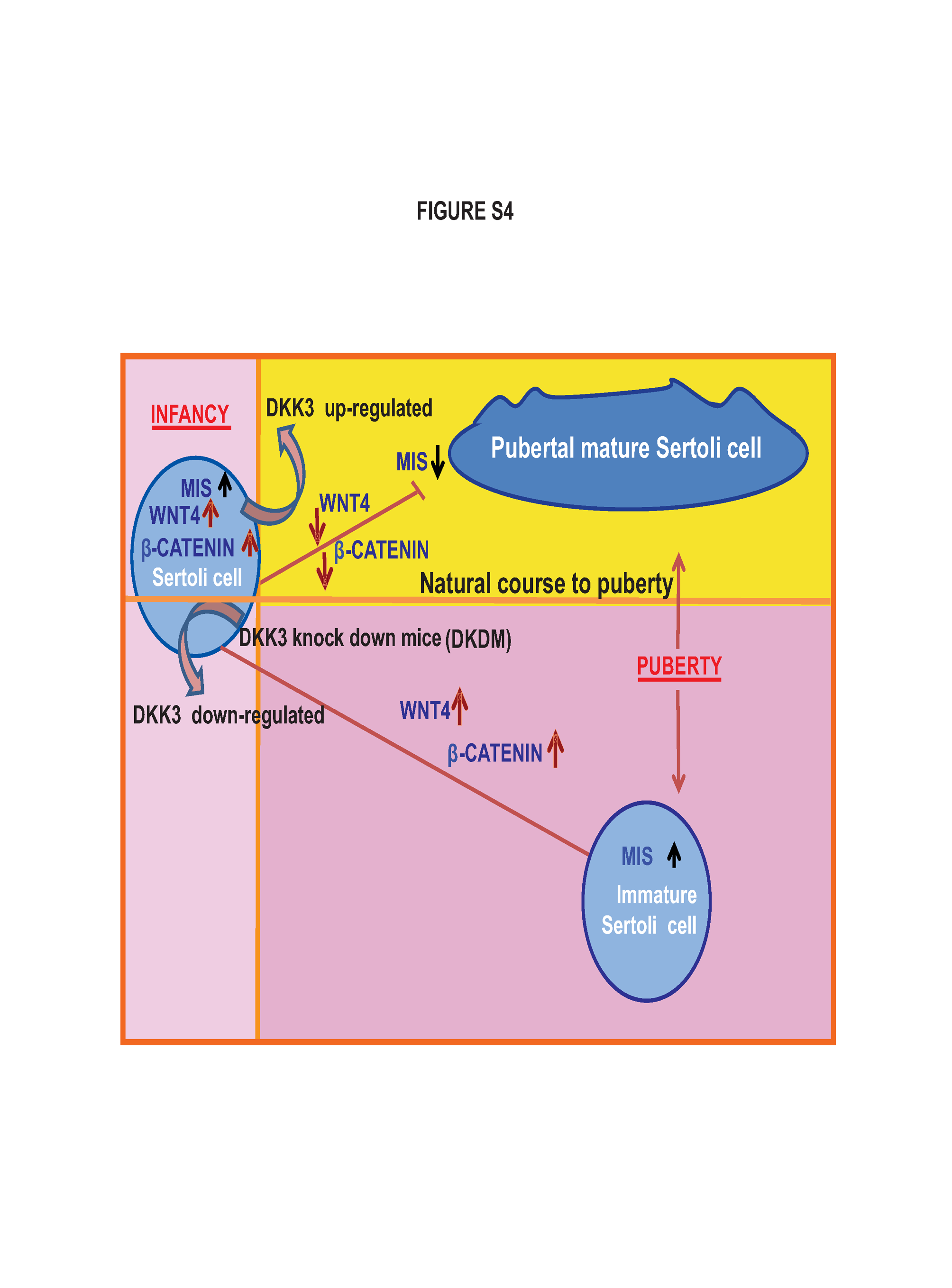

Supplement: Figure S4 — A cartoon showing that upregulation of DKK3 expression during natural course to puberty (yellow section) is responsible for Sc maturation and that inhibition of DKK3 in transgenic DKDM results into maturational failure of Sc (dark pink section). (TIF) [file pone.0063603.s004.tif]
