# Supplementary material for: Dickkopf Homolog 3 (DKK3) Plays a Crucial Role Upstream of WNT/β-CATENIN Signaling for Sertoli Cell Mediated Regulation of Spermatogenesis
Source: PLoS One. 2013 May 7;8(5):e63603. doi: 10.1371/journal.pone.0063603 (PMC3647036; doi:10.1371/journal.pone.0063603)
Supplement: Table S1 — List of primers used for Real time PCR. (DOC) [file pone.0063603.s005.doc]

**Table S1: Primers used for Real time PCR.**

| **Gene** | **Gene ID** | **Forward Primer Sequence**  **5’ to 3’** | **Reverse Primer Sequence**  **5’ to 3’** | **Tm (°C)** |
| --- | --- | --- | --- | --- |
| Monkey ***DKK3*** | 701549 | GAGATGTTCCGCGAGGTTGA | ATAGCTGGGAGGTAAGTTTGC | 66 |
| Monkey ***Cyclophilin A*** | 256374 | AGGTCCTGGCATCTTGTCCA | TGCCAAAGACCACATGCTTG | 62 |
| Mice ***DKK3*** | 50781 | TCCCTTTCTGGCTAACAGGA | ACCAAAGCTGCAGAAGTCTC | 62 |
| ***MIS*** | 11705 | CAGAACCTCTGCCCTACTCG | CACCTTCTCTGCTTGGTTGA | 62 |
| ***WNT4*** | 22417 | ACTGGACTCCCTCCCTGTCT | TGAGAAGGCTACGCCATAGG | 62 |
| ***WNT1*** | 22408 | GCAAGGCCAGGCAGGCCATG | CACTCACGCTGTGCAGGATC | 62 |
| ***WNT3a*** | 22416 | CGATGGCTCCTCTCGGATAC | TGCTGACGGTGGTGCAGTTC | 62 |
| ***WNT8a*** | 20890 | GCAGGACCATGGGACACTTG | GAAGGATGTCTCTCTCGTGG | 62 |
| ***WNT5a*** | 22418 | GGAAGGTGGGCGATGCCCTC | TGCAATGACAGCGTTCGGTC | 62 |
| ***WNT5b*** | 22419 | GCAAGGTGGGGGACCGTTTG | CACCTGAACGCTCTTGAAGC | 62 |
| ***WNT6*** | 22420 | ACGGCTGCTGGAGCGCTTCC | TCTCCTCGAGCTGTACGCTC | 62 |
| ***WNT11*** | 22411 | CTGACCTCAAGACCCGCTAC | CCACCACTCTGTCCGTGTAG | 62 |
| ***β-CATENIN*** | 12387 | CTTGGCTGAACCATCACAGA | TGTCAGCTCAGGAATTGCAC | 62 |
| ***CYP17*** | 13074 | GTGGGTCTCTTGCTGCTCAT | GTCTGGGGAGAAACGGTAGA | 64 |
| Mice ***Cyclophilin A*** | 268373 | ATGGCAAATGCTGGACCAAA | TGCCTTCTTTCACCTTCCCA | 62 |
